# Genetic Diversity in Coppice Chestnut Forests in Central Italy and Potential Use of SSR-Based Timber Traceability

**DOI:** 10.3390/plants15132066

**Published:** 2026-07-02

**Authors:** Martina Marcomeni, Anna Rita Paolacci, Francesco Carbone, Elena Kuzminsky, Mario Ciaffi

**Affiliations:** Department for Innovation in Biological, Agro-Food and Forestry Systems, University of Tuscia, Via S. Camillo de Lellis, I-01100 Viterbo, Italy; martina.marcomeni@unitus.it (M.M.); arpaolacci@unitus.it (A.R.P.); fcarbone@unitus.it (F.C.)

**Keywords:** *Castanea sativa* Mill., simple sequence repeat (SSR) markers, population genetics, forest conservation and management, timber origin verification, forest traceability

## Abstract

Sweet chestnut (*Castanea sativa* Mill.) coppice forests are important Mediterranean resources, but the genetic structure of local coppice stands remains insufficiently characterized, limiting their use as reference systems for wood provenance studies. This study integrated population genetic analysis, optimization of wood-derived DNA recovery, and reference-based assignment to evaluate Lazio coppice stands and test the declared origin of timber samples. Four Lazio populations were genotyped at 12 SSR loci, screened for Hardy–Weinberg disequilibrium, null alleles, and kin structure, and compared with eight European/Mediterranean populations using five shared loci. DNA extraction from dried wood was optimized through a CTAB-PEG workflow, and 40 timber samples from four sawmills, including two declared from Lazio (MCt and RPt), one from Calabria (CALt) and one from France (FRAt), were assigned using supervised DAPC, STRUCTURE with population information and GDA_NT Bayesian assignment and exclusion testing. Lazio stands retained high SSR diversity but showed heterozygote deficiency and fine-scale family structure; therefore, a conservative post-COLONY dataset was used for downstream analyses. Differentiation was weak among Lazio stands, but they showed a coherent Central Italian affinity within the available European/Mediterranean reference set. The optimized protocol yielded reproducible SSR profiles from all timber samples, with an 80.0% successful wood extraction rate. Within the available reference panel, assignment analyses indicated compatibility of MCt and RPt with a Lazio origin, whereas CALt and FRAt were incompatible with the Lazio references and should be interpreted as extra-regional or insufficiently represented by the current baseline. SSRs provide a practical first-line tool for regional chestnut timber screening, although broader reference panels and complementary high-resolution markers are needed to strengthen fine-scale provenance inference.

## 1. Introduction

Sweet chestnut (*Castanea sativa* Mill.) is one of the most important broadleaved tree species in Europe, where it has long provided fruit, durable wood, and multiple ecosystem services. It is cultivated in specialized orchards and managed in coppice systems, occupying more than 2.5 million hectares across Europe [1,2]. Its ecological plasticity, high-quality timber, and long history of human use have promoted extensive cultivation and movement of plant material since prehistoric and Roman times, contributing to the development of numerous local varieties, ecotypes, and managed forest systems throughout the Mediterranean region [3,4].

Molecular markers have substantially improved our understanding of the natural and human-mediated history of *C. sativa*. Multilocus SSR studies have revealed strong geographic structure across Europe, identified major glacial refugia, and highlighted the role of post-glacial expansion and human dispersal in shaping present-day genetic patterns [5,6]. Central Italy is considered an important component of the species’ refugial and diversity landscape [5,6]. SSR markers have also supported cultivar identification, germplasm conservation, and European reference databases, providing a useful framework for comparative genetic analyses and potential origin verification [7,8,9].

In Italy, chestnut forests represent a major forest resource, covering approximately 800,000 ha, with a large proportion managed as coppice stands; Lazio hosts about 36,000 ha of chestnut forest, including extensive coppice systems used primarily for wood production [10]. These stands occur mainly in the Cimini, Sabatini, Alban, and Ernici mountain systems and are embedded in landscapes with long histories of chestnut management. Despite their economic, ecological, and cultural relevance, the genetic diversity and fine-scale structure of Lazio coppice chestnut stands remain insufficiently characterized. Such information is needed to support conservation-oriented management, maintain adaptive potential under environmental change, and establish reliable reference datasets for downstream provenance analyses [11,12].

Genetic traceability is increasingly used as an independent biological tool to support timber origin verification and to complement document-based supply-chain controls [13,14]. However, reliable geographic origin testing is more demanding than species identification. It depends on spatial genetic structure, marker resolution, and the density and representativeness of reference datasets [15,16]. For this reason, international initiatives such as WorldForestID are building standardized geo-referenced wood reference collections to improve the evidential value of timber origin testing [17]. DNA-based approaches have already shown potential for several timber species, but their accuracy remains strongly constrained by baseline data quality and coverage [18,19]. The application of genetic methods to timber is technically demanding. 

Dried and lignified wood usually contains little fragmented endogenous DNA and many co-extracted PCR inhibitors [20,21]. Reliable SSR-based timber testing therefore requires optimized DNA extraction, strict contamination control, reproducibility checks, and explicit statistical assignment procedures. These requirements are relevant in the European regulatory context, where the EU Timber Regulation and EU Deforestation Regulation reinforce due-diligence obligations and the need for tools that can support origin verification [22,23].

In sweet chestnut, few studies have integrated regional population genetic characterization, optimization of DNA extraction from dried wood, SSR genotyping of timber samples, and formal reference-based assignment within the same framework. This gap is particularly relevant for coppice systems, where vegetative persistence, local recruitment, and family structure may influence allele-frequency estimates and therefore affect population structure and assignment analyses. Moreover, assignment of timber samples requires a clear distinction between descriptive analyses of genetic structure and formal testing of unknown samples against reference populations. This distinction is essential when evaluating whether timber is compatible with a declared origin or whether the true source may be absent from the available reference panel.

The present study addresses these issues using coppice chestnut stands from four areas of the Lazio region and timber samples obtained from two Lazio sawmills and two extra-regional sources. Specifically, we aimed to: (i) characterize genetic diversity and population structure in Lazio coppice chestnut stands using 12 nuclear SSR loci; (ii) assess Hardy–Weinberg equilibrium (HWE) departures, possible null-allele effects, and within-stand kin structure, and use this information to define a conservative reference dataset; (iii) compare the Lazio populations with published SSR profiles from eight European and Mediterranean populations [24]; (iv) optimize DNA extraction and SSR amplification from dried chestnut wood; and (v) evaluate timber assignment at regional and European/Mediterranean scales using reference-based DAPC, STRUCTURE, and Bayesian assignment and exclusion testing. 

By integrating population genetic analysis, wood-derived DNA optimization, and formal assignment methods, this study provides a scientific basis for the conservation and management of local Lazio coppice chestnut forests. It also evaluates, within a preliminary but operational framework, the potential of SSR markers for regional timber provenance verification. The approach is intended to support local forest-based value chains and transparent timber supply systems while recognizing that fine-scale assignment requires broader reference datasets.

## 2. Materials and Methods

### 2.1. Sampling Sites, Plant Materials, Reference Populations, and Sample Preparation

#### 2.1.1. Leaf Tissues

The selected sites are in four chestnut-growing areas of Lazio (Central Italy) characterized by extensive coppice stands in the Cimini, Sabatini, and Ernici Mountains and in the Alban Hills. Leaf sampling was carried out along a northwest–southeast transect in the municipalities of San Martino al Cimino (560 m a.s.l.; 42.370° N, 12.128° E), Oriolo Romano (420 m a.s.l.; 42.157° N, 12.137° E), Rocca di Papa (680 m a.s.l.; 41.770° N, 12.703° E), and Fiuggi (747 m a.s.l.; 41.799° N, 13.221° E) (Table 1, Figure S1). The first three areas are historical hubs for chestnut wood production and processing [25], whereas Fiuggi represents a different management context, where chestnut forests have a prominent protective role, particularly in safeguarding local water resources [26]. Hereafter, the study sites are referred to as IT02, IT03, IT04, and IT05, respectively. To assess the genetic distinctiveness and discriminative power of these Lazio populations within a broader European context, genotypic data for five of the twelve SSR loci were retrieved from [24] for eight additional European chestnut populations: three from Spain, two from Italy, two from Greece, and one from Turkey (Table 1). At each Lazio site, six leaves were collected from a single shoot of each of 40 randomly selected stools, which were spaced at least 30 m apart and located away from edge effects. Samples were transported under refrigeration and stored at −80 °C until DNA extraction.

#### 2.1.2. Wood Samples

The timber sampling design included two Lazio sawmills, located in San Martino al Cimino and Rocca di Papa, which supplied certified material from the forest stands investigated. For comparison, two extra-regional sawmills were included: one in Southern Italy (Fabrizia, Province of Vibo Valentia, Calabria) and one in France (Pays de la Loire Region, Sarthe Department, Le Mans area). Timber sources were labeled MCt, RPt, CALt, and FRAt. Ten logs were randomly selected per sawmill, and one wood disk was collected from each log to account for within-sawmill variability. Disks were selected according to strict quality criteria, including the presence of sapwood and the absence of rot. Before processing, bark and external surface layers were removed with sterile blades. Sapwood shavings (100–200 mg) were collected from the inner disk using sterile drill bits or a micro-planer and stored in 2 mL tubes. Tools were decontaminated with 10% bleach and 70% ethanol between samples. Material was ground in liquid nitrogen with pre-chilled mortars and stored at −20 °C until DNA extraction.

### 2.2. Extraction, Evaluation, and Quality Control of DNA

#### 2.2.1. Leaf Tissues

Total genomic DNA was extracted from 168 leaf samples (40 individuals from each of the four populations plus eight controls) using the NucleoSpin^®^ Plant II kit (Macherey-Nagel, Düren, Germany), according to the manufacturer’s instructions. DNA quality and concentration were assessed by 1.2% (*w*/*v*) agarose gel electrophoresis and a NanoDrop ND-1000 spectrophotometer (Thermo Fisher Scientific, Waltham, MA, USA). Samples were normalized to 20 ng/µL and stored at −20 °C until use.

#### 2.2.2. Wood Samples

Because wood contains low amounts of endogenous, often degraded DNA and PCR-inhibiting compounds, several extraction procedures were initially evaluated. These included a conventional CTAB protocol, phenol–chloroform extraction, commercial plant DNA kits NucleoSpin® Plant II kit (Macherey-Nagel GmbH & Co. KG, Düren, Germany), DNeasy Plant Mini Kit and DNeasy PowerPlant Pro Kit (QIAGEN, Hilden, Germany) and a hybrid CTAB/silica-column protocol. Trials were performed with 100 mg to 5 g of wood powder and were modified by extending lysis, increasing buffer volumes, and adding 2.5% Polyvinylpyrrolidone (PVP; Sigma-Aldrich, St. Louis, MO, USA) to reduce polyphenol-related inhibition. However, these approaches generally yielded low amounts of DNA, often not clearly detectable by agarose gel electrophoresis, consistent with the expected degradation and low abundance of DNA in wood.

Based on these trials, a modified CTAB protocol [27] combined with PEG purification was selected. Briefly, 100 mg of wood powder was incubated in CTAB buffer for 5 h, extracted with chloroform-isoamyl alcohol, and precipitated overnight at −20 °C with isopropanol. The pellet was washed with 70% ethanol, dried, and resuspended in 50 µL of water. Residual inhibitors were removed using an NH4OAc/PEG purification step: samples were adjusted to 100 µL, mixed with an equal volume of NH_4_OAc, incubated on ice for 1 h, and centrifuged for 20 min; the supernatant was then precipitated with 200 µL of PEG 8000 (Sigma-Aldrich, St. Louis, MO, USA) for 1 h on ice, centrifuged, washed, vacuum-dried, and resuspended in 50 µL of water. For each of the 40 timber samples, three independent DNA extractions were performed from separate aliquots of wood powder. Although physically separated rooms were not available, fresh leaf and timber materials were processed in temporally separated working sessions. Work surfaces and equipment were decontaminated before and after each session, aerosol-resistant filtered tips and dedicated or freshly aliquoted reagents were used, and negative extraction controls were included throughout the procedure.

Each independent wood extraction was evaluated using complementary spectrophotometric and fluorometric measurements. Spectrophotometric analyses were performed with a NanoDrop ND-1000, recording DNA concentration and the A260/A280 and A260/A230 absorbance ratios. Double-stranded DNA was quantified with a Qubit™ Flex Fluorometer (Thermo Fisher Scientific, Waltham, MA, USA) using the Qubit dsDNA HS Assay Kit (Thermo Fisher Scientific, Waltham, MA, USA). For each extraction, 10 µL of the 50 µL DNA eluate was diluted 1:5 with Milli-Q water. Two technical replicate measurements were performed on both platforms using 5 µL of the diluted aliquot, and mean values were used for interpretation and reporting. In parallel, 16 leaf DNA samples from previously SSR-genotyped individuals (four from each Lazio population: IT02, IT03, IT04, and IT05) were analyzed as reference controls and used for SSR allele standardization in timber samples.

### 2.3. Genetic Analysis Using SSR Molecular Markers

#### 2.3.1. Leaf Tissues

Genetic variability within and among chestnut populations from four representative areas of the Lazio region was assessed using 12 nuclear SSR loci [28,29] selected for high polymorphic information content (PIC) and genome coverage (Table S1). 

Preliminary amplifications with non-fluorescent primers were performed on randomly selected DNA samples to verify primer specificity and optimize PCR conditions; products were checked on 1.2% (*w*/*v*) agarose gels stained with ethidium bromide.

Final PCR reactions were carried out in 25 µL volumes containing 20 ng of genomic DNA, 0.4 µM of each primer, and 12 µL of GoTaq^®^ Hot Start Colorless Master Mix (Promega Corporation, Madison, WI, USA). Forward primers were fluorescently labeled with 6-FAM, HEX, or TAMRA. Thermal cycling consisted of 94 °C for 2 min; 35 cycles of 94 °C for 30 s, locus-specific annealing at 50–59 °C (Table S1) for 30 s, and 72 °C for 1 min; followed by a final extension at 72 °C for 5 min. Amplicons were separated on an ABI PRISM^®^ 3500 Genetic Analyzer (Applied Biosystems, Foster City, CA, USA), available at the C.I.B.I.A.C.I. Service Center, and allele sizing was performed in GeneMapper^®^ v4.1 software (Applied Biosystems, Foster City, CA, USA) using the ROX 400 size standard. Eight DNA samples with known SSR profiles from the CNR-IRET Institute (Porano, TR, Italy) were included as internal calibration controls to ensure inter-laboratory allele-size comparability.

#### 2.3.2. Wood Samples

The 40 timber samples were amplified at the seven most informative and reproducible SSR loci selected from the full marker set: CsCAT1, CsCAT2, CsCAT3, CsCAT6, CsCAT16, CsCAT17, and EMCs38. PCR conditions were the same as for leaf-derived DNA, except for two modifications introduced to improve amplification from wood-derived DNA: bovine serum albumin (BSA; Sigma-Aldrich, St. Louis, MO, USA) was added at 0.5 µg/µL to reduce inhibition by residual wood compounds, and HOT FIREPol^®^ DNA Polymerase (Solis BioDyne, Tartu, Estonia) was used instead of GoTaq because it showed higher amplification efficiency in preliminary tests. PCR set-up for wood-derived DNA was conducted in working sessions separated from those used for leaf DNA, using decontaminated work surfaces and filtered tips. Extraction blanks and non-template PCR controls were included in each batch to monitor contamination.

To assess the reproducibility of wood-derived SSR profiles, a random subset of timber samples was analyzed using at least two DNA preparations obtained from independent extractions of the same wood sample and amplified at all seven SSR loci. This quality control step verified fragment-profile reproducibility and electrophoretic peak quality rather than systematically re-genotyping all three extracts from each timber sample. PCR products were first checked by agarose gel electrophoresis for amplification success and expected fragment size and then analyzed by capillary electrophoresis. Genotype quality was evaluated by comparing peak profiles and allele calls from independent extracts using the same binning criteria applied to leaf-derived DNA. Under the optimized conditions, replicated wood-derived DNA extracts yielded PCR products of the expected size and interpretable electrophoretic profiles comparable to those obtained from leaf-derived DNA. No systematic evidence of allelic dropout or peak ambiguity was detected. Reliable multilocus SSR profiles were obtained for all 40 timber samples, allowing the final genotypes at the seven selected loci to be incorporated into the downstream statistical and assignment analyses described in Section 2.4 and Section 2.5.

### 2.4. Statistical Data Analysis

#### 2.4.1. Genetic Diversity Parameters of the SSR Loci

For the complete dataset (160 genotypes), alleles at 12 codominant SSR loci were scored by fragment size (bp). Genetic diversity indices were calculated with PowerMarker v.3.25 [30] and GenAlEx v.6.5 [31]. For each locus, the following parameters were estimated: number of alleles (Na), effective number of alleles (Ne), major allele frequency (MAF), Shannon’s information index (I), observed heterozygosity (Ho), expected heterozygosity (He), unbiased expected heterozygosity (uHe), inbreeding coefficient (F), and polymorphic information content (PIC) [32].

#### 2.4.2. HWE-Related, Null-Allele, and Kin-Structure Analyses

Departure from Hardy–Weinberg equilibrium (HWE) was evaluated for each locus within each population using both descriptive and exact approaches. In GenAlEx v.6.5, Ho, He, and Fis were calculated, and chi-square comparisons between observed and expected genotype frequencies were used as an initial screen [31]. Locus-by-population departures from HWE and heterozygote deficits were then tested with Genepop exact tests [33].

To assess whether heterozygote deficiency reflected null alleles, inbreeding, or both, each population was analyzed in INEst2 [34] under the null, n, f, nf, and nfb models. Model support was compared using the deviance information criterion (DIC). Biological interpretation focused on the lowest-DIC model, the inbreeding parameter F when estimated, the b parameter in the nfb model, and locus-specific posterior mean null-allele frequencies (p0). For cross-population comparison, loci with the highest p0 values under the nf model were also recorded because this model is informative for separating null alleles from homozygote excess generated by broader biological structure [34].

Family structure within each population was assessed with COLONY using multilocus sibship reconstruction [35]. Populations were analyzed separately under a monoecious diploid model with male and female polygamy, no candidate parents, and medium-length full-likelihood inference. BestConfig, BestFSFamily, HalfSibDyad, and ConfigArchive outputs were used to identify robust full-sib (FS) groups and recurrent half-sib (HS) links. Conservative and severe datasets were then created by retaining one representative per robust FS group and, for the severe dataset, additionally removing recurrent HS bridge individuals.

#### 2.4.3. Population Structure Analyses and Post-COLONY Dataset Selection

Population structure was evaluated with STRUCTURE v.2.3.4 on the original (N = 160), conservative (N = 100), and severe (N = 73) post-COLONY datasets [36]. For each dataset, 20 independent runs were performed for K = 1–10, with 100,000 burn-in iterations and 500,000 MCMC iterations, using an admixture model with correlated allele frequencies. Population labels were retained for sample tracking, but clustering was unsupervised (USEPOPINFO = 0). STRUCTURE outputs were summarized in STRUCTURE SELECTOR [37] using mean LnP(K), Evanno ΔK [38], and the four Puechmaille estimators [39]. Because the post-COLONY datasets differed in size, each was analyzed separately with a popmap for IT02, IT03, IT04, and IT05; Puechmaille statistics were calculated at thresholds of 0.5, 0.6, 0.7, and 0.8.

Replicate runs were aligned and visualized in CLUMPAK [40] using the default CLUMPP LargeKGreedy algorithm with 2000 repeats. Main and supplementary bar plots were assembled from CLUMPAK/DISTRUCT major-mode outputs, and a supplementary heat map of Puechmaille-selected K values across thresholds was generated from STRUCTURE SELECTOR tables in Microsoft Excel. Dataset selection did not rely on a single criterion but jointly considered (i) agreement among LnP(K), ΔK, and Puechmaille estimators, (ii) replicate stability and CLUMPAK mode consistency, and (iii) biological interpretability with respect to the four sampled populations.

#### 2.4.4. Genetic Structure and Differentiation of the Four Lazio Populations Based on the Conservative Dataset

Because the conservative dataset was the least kin-biased yet still interpretable post-COLONY dataset, it was used for all subsequent within-Lazio analyses. Population structure among IT02, IT03, IT04, and IT05 was summarized at K = 4 from CLUMPAK major-mode membership coefficients. GenAlEx v.6.5 was used to estimate Na, Ne, Npa, I, Ho, He, uHe, and F. Differentiation was assessed by AMOVA and by estimating Fst, standardized G’st [41], Nm, and Nei’s unbiased genetic distance [42]. Significance was tested with 999 permutations, and Nm was calculated as Nm = [(1/Fst) − 1]/4.

#### 2.4.5. Comparative Analysis with European Chestnut Populations

To evaluate the genetic distinctiveness of the Lazio chestnut populations in a broader European context, five SSR loci (CsCAT1, CsCAT3, CsCAT16, CsCAT6, and EMCs38) were selected from the 12 loci analyzed in this study. These loci were chosen because comparable genotypes are available in the literature for eight natural European populations [24], including three from Spain, two from Italy (Piedmont and Sicily), two from Greece, and one from Turkey (Table 1). The four Lazio stands were represented by the conservative post-COLONY dataset.

Population structure across the 12 populations was inferred with STRUCTURE v.2.3.4 using the settings described in Section 2.4.3: admixture model, correlated allele frequencies, 20 runs per K, 100,000 burn-in iterations, and 500,000 MCMC iterations. K values from 1 to 12 were tested without prior population information (USEPOPINFO = 0). Outputs were summarized in STRUCTURE SELECTOR using mean LnP(K), Evanno ΔK, and the four Puechmaille estimators at thresholds of 0.5, 0.6, 0.7, and 0.8; replicate runs were aligned in CLUMPAK. Final interpretation was based on agreement among these criteria, replicate stability, and biological interpretability. On this basis, K = 2 and K = 3 were retained, and mean membership proportions (Q) were calculated from the corresponding CLUMPAK-aligned major modes.

Genetic diversity across the 12 populations was assessed by estimating allelic richness (AR) with FSTAT v.2.9.3.2 [43]. Additional diversity parameters, including Na, Ne, Npa, I, Ho, He, and uHe, were calculated in GenAlEx v.6.5 using the same criteria adopted for the Lazio dataset.

#### 2.4.6. Statistical Analysis of DNA Quality and SSR Amplification Success

Statistical analyses and graphical outputs were generated in JMP^®^ Pro version 15 (SAS Institute Inc., Cary, NC, USA). DNA concentration data were summarized as medians and interquartile ranges and analyzed using non-parametric tests: Wilcoxon signed-rank tests for paired NanoDrop–Qubit comparisons, Wilcoxon rank-sum tests for independent groups, and Fisher’s exact tests for associations between Qubit concentration classes and PCR amplification success. Significance was set at *p* < 0.05.

### 2.5. Reference-Based Genetic Assignment of Timber Samples

#### 2.5.1. Assignment Datasets and Reference Panels

Formal assignment analyses tested the genetic compatibility of timber genotypes with predefined reference populations, independently of the exploratory population structure analyses described above. Reference individuals were used to train or estimate the assignment models, whereas timber genotypes were treated as external samples of unknown origin.

For the four Lazio reference populations, assignment analyses used the conservative post-COLONY dataset. Two datasets were analyzed. The Lazio assignment dataset included 100 reference individuals from IT02, IT03, IT04, and IT05, plus 40 timber samples from four sawmills (MCt, RPt, CALt, and FRAt; 10 samples each), genotyped at seven SSR loci: CsCAT1, CsCAT2, CsCAT3, CsCAT6, CsCAT16, CsCAT17, and EMCs38. The European/Mediterranean dataset included 309 reference individuals from 12 populations, including the same four Lazio populations and eight additional European and Mediterranean populations, together with the same 40 timber samples genotyped at five shared SSR loci: CsCAT1, CsCAT3, CsCAT16, CsCAT6, and EMCs38.

#### 2.5.2. Supervised DAPC Assignment

Discriminant analysis of principal components (DAPC) was performed using the adegenet package v2.1.3 in R v4.0. (R Foundation for Statistical Computing, Vienna, Austria), following the methodology described by [44] and further developed for the analysis of genetically structured populations [45,46].

DAPC was implemented as a supervised reference-based assignment procedure. Genotype tables were converted to genind objects, reference and timber samples were separated before model fitting, and reference population labels were used as predefined groups. Timber samples were excluded from the construction of discriminant functions and subsequently projected onto the reference discriminant space using predict.dapc.

Analyses were performed independently for the two datasets. In the Lazio dataset, IT02, IT03, IT04, and IT05 were used as reference groups, and three discriminant functions were retained. In the European/Mediterranean dataset, the 12 reference populations were used as predefined groups, and 11 discriminant functions were retained. The number of principal components was selected with xvalDapc by testing up to 50 PCs, using 90% of reference individuals for training, 100 cross-validation replicates, centered but unscaled allele-frequency data, and assignment success as the optimization criterion. For each timber sample, posterior membership probabilities were extracted from predict.dapc, and the group with the highest posterior probability was recorded as the most likely assignment. Reference-panel performance was evaluated using confusion matrices comparing known reference labels with DAPC-predicted classes.

#### 2.5.3. STRUCTURE Assignment Using Population Information

Reference-based Bayesian assignment was performed using STRUCTURE v.2.3.4 [36,47], separately from the exploratory STRUCTURE analyses used to describe population structure. Prior population information was used only for reference individuals: reference samples were coded with POPFLAG = 1, whereas timber samples were coded with POPFLAG = 0 and treated as unknown. Analyses were run with USEPOPINFO = 1 and PFROMPOPFLAGONLY = 1 so that allele frequencies were estimated only from reference individuals with known origin.

The admixture model with correlated allele frequencies was used. For each dataset, 20 runs were performed with 100,000 burn-in iterations and 500,000 MCMC iterations. K was fixed to the number of tested reference populations: K = 4 for the Lazio dataset and K = 12 for the European/Mediterranean dataset. MIGRPRIOR was set to 0.05 and GENSBACK to 2. For each timber sample, membership coefficients (Q-values) were averaged across runs. The reference population or group with the highest mean Q-value was recorded as the most likely STRUCTURE affinity, whereas low or broadly distributed Q-values were interpreted as weak or uncertain assignment.

#### 2.5.4. Bayesian Assignment and Exclusion Testing with GDA_NT 2021

Bayesian assignment and exclusion tests were performed using GDA_NT 2021 [48], with separate reference and timber input files for each dataset. In the main analysis, the 40 timber samples were treated as 40 independent test groups, each containing one individual, to evaluate traceability performance at the individual level. Reference-panel discriminatory power was first assessed by leave-one-out self-assignment of reference individuals with the group size set to 1. Timber assignment was then performed using the Bayesian method of [49]. All SSRs were treated as biparentally inherited codominant markers, and the minimum number of complete biparental loci required for assignment was set to seven for the Lazio dataset and five for the European/Mediterranean dataset. Exclusion probabilities were calculated using 10,000 simulated groups, with reference allele frequencies corrected by F-values and by the 99% confidence interval of allele frequencies.

For each timber sample, the population with the lowest −log10 genotype likelihood was considered the most likely population among those available in the reference panel. This relative assignment was considered statistically supported only when the corresponding exclusion probability was below the 95% exclusion threshold; probabilities ≥ 0.95 were interpreted as relative affinity rather than reliable population-level assignment. Samples excluded from all reference populations were considered genetically incompatible with the available panel, suggesting that the true source population was absent or insufficiently represented.

Individual-level assignment was the primary output for DAPC, STRUCTURE, and GDA_NT. Sawmill-level summaries were calculated only after individual assignment by summarizing the 10 samples collected from each sawmill. As a supplementary control, GDA_NT was also run with four sawmill-level test groups of 10 individuals each; this analysis evaluated within-source consistency and was not considered the primary measure of traceability performance. Integrated interpretation was based on concordance among DAPC posterior probabilities, STRUCTURE Q-values, likelihood-based best matches, and exclusion probabilities.

## 3. Results and Discussion

### 3.1. Genetic Diversity of Lazio Chestnut Coppice Stands Analyzed Using 12 SSR Loci

All 12 SSR loci were polymorphic in the complete Lazio dataset (160 individuals), yielding 110 alleles and a mean of 9.17 alleles per locus (Table 2). Across the four populations, 13 private alleles were detected, 12 of which were rare (<5% frequency; Table S2). Mean Ne, major allele frequency, Shannon’s information index, and PIC were 4.301, 0.354, 1.599, and 0.737, respectively, confirming the high informativeness of the marker set. Observed heterozygosity was consistently lower than expected heterozygosity (Ho = 0.516; He = 0.738; uHe = 0.748), and mean F was positive (0.302), indicating multilocus homozygote excess.

The Lazio coppice stands retained substantial SSR diversity despite their restricted geographic range and long management history. Mean allelic diversity and He were within the range reported for broader European and Mediterranean datasets [9,50,51], and allelic richness was comparable to or higher than values reported for several local or germplasm-based surveys [52,53,54,55,56]. The high mean PIC, particularly at EMCs38, CsCAT2, CsCAT17, and CsCAT3, confirmed the discriminatory value of the marker set [51,52,57].

### 3.2. HWE Departure, Null Alleles, and Family Structure

Departure from HWE was widespread across the four populations and was consistently associated with heterozygote deficiency. In all populations, most loci showed lower Ho than He and positive mean Fis values (Table S3). Genepop exact tests confirmed significant HWE departure at 8–10 loci per population, indicating a population-wide signal rather than a deviation driven by only a few markers.

At the locus level, EMCs25 and EMCs32 showed the strongest and most recurrent deviation, combining low mean Ho, high mean Fis, and significance in all four populations (Table S4). CsCAT41, CsCAT2, and CsCAT34 formed a second consistently deviant group, whereas CsCAT1, CsCAT14, and CsCAT17 contributed little to the overall pattern.

INEst2 refined but did not overturn this interpretation (Table S5). The f model had the lowest DIC in IT02 and IT05, suggesting a dominant inbreeding-like component. In IT03 and IT04, the n model had the lowest DIC; however, this result should be interpreted cautiously because COLONY later revealed strong within-stand kin structure, and the nf model remained close to the best fit. Thus, INEst2 supported a secondary, locus-specific null-allele component superimposed on broader biological structure rather than a purely technical explanation. The nfb model did not substantially improve fit, and mean b values remained low, indicating that generalized genotyping failure was not the main driver. The main candidate loci for null-allele effects were EMCs32, EMCs25, CsCAT2, CsCAT41, CsCAT34, and EMCs38, with the highest nf-based p0 estimates in IT03 and IT04.

COLONY confirmed that family structure was pervasive and represented a major biological component of the observed HWE departure (Table S6). Robust full-sib groups ranged from four in IT03 to six in IT02 and IT04. Retaining one representative per robust full-sib group reduced sample size by 30.0–45.0% in the conservative datasets, whereas severe pruning removed 40.0–62.5% of individuals. Close kin were therefore sufficiently common to influence allele-frequency estimates and downstream analyses relying on within-group HWE assumptions.

Comparison of the original, conservative, and severe datasets showed that COLONY-based filtering caused only a modest loss of diversity (Table 3). Total alleles decreased from 110 to 106 and 104 in the conservative and severe datasets, respectively, and mean Na declined from 9.17 to 8.83 and 8.67. Frequency-based descriptors remained stable or increased slightly, with mean Ne, He, Shannon’s information index, and PIC showing marginal changes after filtering. The clearest biological effect concerned heterozygosity and inbreeding: mean Ho increased from 0.516 to 0.526 and 0.524, while mean F decreased from 0.331 to 0.316 and 0.322 in the conservative and severe datasets. The strongest locus-level reductions in F occurred at EMCs25, EMCs32, and EMCs38, which also showed the clearest increases in Ho. Thus, kin aggregation contributed substantially to the original homozygote excess, although it was not the only factor involved.

Taken together, HWE tests, INEst2, and COLONY indicate that within-stand family structure was the main driver of multilocus homozygote excess, with null alleles contributing as secondary, locus-specific effects. The recurrent involvement of EMCs32, EMCs25, CsCAT2, CsCAT41, CsCAT34, and EMCs38 supports possible marker-specific behavior. This interpretation is consistent with INEst2, which can overestimate null-allele frequencies when homozygote excess is produced by inbreeding or broader biological structure [34], and with the distinction between Wahlund effects and null alleles discussed by [58].

This pattern is biologically plausible in long-managed coppice stands. Repeated cutting favors stool persistence and vegetative resprouting, so stands may function as mosaics of stool-centered kin neighborhoods rather than panmictic units. Pooling such neighborhoods can generate Wahlund-like heterozygote deficits, whereas spatially restricted mating and uneven reproductive success can maintain full-sib and half-sib clusters. The recurrent COLONY groups are therefore consistent with stool longevity, local recruitment, and family clustering in coppice systems [59,60].

These findings are consistent with forest-tree studies showing that heterozygote deficits may have both biological and marker-specific causes [61,62,63,64,65,66]. Clean electropherograms do not exclude null alleles because primer-site mutations or preferential amplification failure can convert true heterozygotes into apparent homozygotes [65,66]. In the present dataset, however, such effects did not appear to be the dominant source of disequilibrium.

Overall, the dataset combines high SSR diversity, widespread heterozygote deficiency, positive Fis values, and strong within-stand kin structure, with possible secondary null-allele effects. The detailed diagnostic outputs are provided in Tables S3–S6. These results justified retaining all 12 loci for STRUCTURE and using the conservative and severe COLONY-filtered datasets as sensitivity tests of clustering stability after kin reduction.

### 3.3. Population Structure After COLONY Filtering and Dataset Selection

Across the original, conservative, and severe post-COLONY datasets, model-selection criteria did not converge on a single K (Table S7; Figure S2). Increasing kin filtering also progressively weakened the inferred structure. The original dataset produced the strongest signal, but it was also most likely to retain kin-driven inflation. The conservative dataset retained an interpretable pattern after reducing the strongest full-sib and half-sib components, whereas the severe dataset showed diffuse ancestry coefficients.

**Figure 1:**
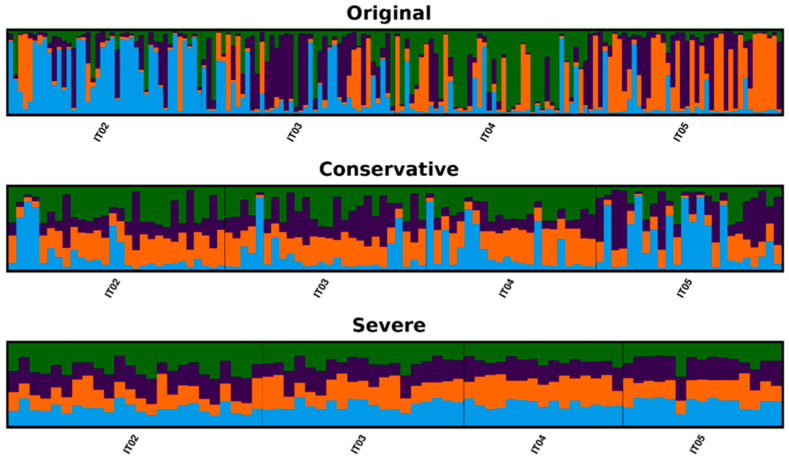
CLUMPAK/DISTRUCT ancestry barplots at K = 4 for the original, conservative, and severe datasets. Panels were assembled from the original CLUMPAK/DISTRUCT export files for the major mode after CLUMPP alignment, with individuals ordered by sampled population (IT02, IT03, IT04, IT05). The original dataset retains the sharpest population-associated assignments, the conservative dataset preserves a weaker but still interpretable pattern, and the severe dataset shows substantial flattening of ancestry profiles.

CLUMPAK supported this interpretation (Table S7; Figure 1). At K = 4, the original dataset showed the sharpest population-associated profiles; the conservative dataset showed a weaker but still interpretable pattern; and the severe dataset was stable but less informative. Thus, the comparison emphasized the robustness of the general signal rather than the definitive biological meaning of any single K value.

Because the four sampled stands correspond to four predefined populations, K = 4 was retained as a useful working representation for within-Lazio comparisons, not as a definitive estimate of the number of biological units. At this K, the original dataset retained the sharpest population-associated profiles but also the greatest risk of residual family aggregation. The conservative dataset preserved a weaker yet non-random pattern after kin filtering, whereas the severe dataset approached near-uniform admixture.

The K = 5 solution did not improve interpretation (Table S8; Figure S3). In all datasets, mean maxQ was lower at K = 5 than at K = 4 (original: 0.582 vs. 0.762; conservative: 0.446 vs. 0.500; severe: 0.292 vs. 0.343), indicating that the additional cluster mainly redistributed ancestry rather than clarifying population boundaries. The Puechmaille heat map (Figure S4) also showed no consistent shift toward a more informative higher K.

Accordingly, the conservative dataset was selected as the primary dataset for downstream analyses. This choice balanced kin reduction and retained resolution while recognizing that STRUCTURE outputs should be interpreted as summaries of weak genetic structure rather than as definitive cluster counts. This interpretation is consistent with evidence that related individuals can inflate STRUCTURE-based clustering and affect K inference [39,67]. The sensitivity analyses are reported in Tables S7 and S8 and Figures S2–S4.

### 3.4. Genetic Structure and Differentiation of the Four Lazio Populations Based on the Conservative Dataset

After selection of the conservative post-COLONY dataset as the primary dataset (Section 3.3; Table S7; Figure 1), within-Lazio structure was summarized at K = 4 to compare the four sampled stands. Mean membership coefficients indicated extensive shared ancestry, with no population dominated by a single cluster (Table 4). IT05 showed the highest contribution from Cluster 1 (Q = 0.354), IT04 from Cluster 2 (Q = 0.311), IT02 from Cluster 4 (Q = 0.308), and IT03 showed the most even distribution across Clusters 2–4. Thus, after reducing kin-related bias, the conservative dataset retained the broad pattern of high admixture and modest population-specific shifts observed in the original dataset. 

This pattern is biologically plausible for chestnut coppice systems. As an obligately outcrossing species, sweet chestnut can maintain gene flow through synchronous flowering and effective pollen and seed dispersal when strong barriers are absent [6]. Weak regional structuring also agrees with the shared history and connectivity of Central Italian chestnut stands [5,6,68], and traditional coppice management may have further preserved diversity and exchange among stands [6,69].

The COLONY results remain relevant to this interpretation. Full-sib and half-sib groups were pervasive within stands, confirming that fine-scale family structure is a real component of the sampled populations and can inflate clustering and heterozygote deficits when close relatives are sampled together. However, because the same general ancestry pattern persisted after conservative kin filtering, family structure appears to amplify rather than generate the signal *de novo*.

AMOVA supported weak but significant differentiation (Table 5): 3.7% of the total variance occurred among populations and 96.3% within populations. Overall differentiation was low but significant (Fst = 0.037, *p* = 0.001), and the indirect estimate of gene flow remained high (Nm = 6.519). Because highly polymorphic SSR loci can depress classical Fst values, standardized G’st was also calculated; the resulting value (G’st = 0.219; Table 5) indicates detectable differentiation once marker polymorphism is considered, although most variation remains within populations.

Pairwise comparisons further supported this pattern (Table S9). IT02 and IT03 were the closest pair, showing the lowest Fst (0.022), standardized G’st (0.154), and Nei’s unbiased genetic distance (0.073), together with the highest inferred Nm (11.055). The strongest differentiation occurred between IT04 and IT05, which showed the highest Fst (0.034), G’st (0.246), and Nei’s distance (0.192), and the lowest Nm (7.167). IT05 also tended to be more differentiated from IT02 and IT03 than these populations were from one another, but all pairwise values remained modest, consistent with the admixed STRUCTURE profiles.

Population-level diversity remained high across all four stands after filtering (Table S10). Expected heterozygosity ranged from 0.696 in IT04 to 0.765 in IT05, and the effective number of alleles from 3.684 in IT04 to 4.783 in IT05. Private alleles were retained in every stand, from one in IT04 to five in IT05. IT02 showed the highest observed heterozygosity (Ho = 0.565) and the lowest inbreeding coefficient (F = 0.211), whereas IT05 showed the highest He, uHe, Shannon’s index, and F value. These results indicate that kin filtering reduced the influence of family structure without erasing the high standing diversity detected in the complete dataset.

The He values observed here are comparable to those reported for Central Italian chestnut populations analyzed with partly overlapping SSR panels, including the 0.58–0.80 range reported by [6]. Because that study identified high genetic diversity in Central Italy, the presence of relict ancient genotypes in the Lazio stands cannot be excluded. The present He and Ne values are also consistent with, or higher than, values reported for other European chestnut populations, including datasets from Spain, Italy, France, Bulgaria, Bosnia and Herzegovina, Croatia, and the western Balkans [5,6,62,69,70,71].

Positive F values across all populations indicate persistent heterozygote deficits, plausibly linked to fine-scale family structure, vegetative regeneration, and spatial clustering of related stools. At the same time, the persistence of private alleles, relatively high Ne, and consistently high He values indicates that none of the four stands is genetically depauperate. The coexistence of high within-population diversity and weak among-population differentiation suggests that each stand retains a substantial fraction of the broader regional diversity while differing only modestly in allele-frequency composition.

Overall, the conservative dataset supports the same general interpretation as the original dataset, but on a less kin-biased basis. High admixture, weak differentiation, and the predominance of within-population variation are unlikely to be artifacts produced solely by within-stand relatedness. Instead, they point to a connected regional gene pool in which family structure acts mainly within stands, whereas differentiation among stands remains limited but significant. From a conservation perspective, the combination of high within-population variation, private alleles, and weak regional structuring suggests that Lazio chestnut coppice stands retain substantial adaptive potential within a shared regional gene pool.

### 3.5. Genetic Diversity and Structure of Lazio and European Chestnut Populations

As a second objective, this study assessed whether a reduced five-locus SSR panel could place the Lazio chestnut populations within a broader European context and support the interpretation of timber provenance. Published genotypes for eight European populations of *C. sativa*, comprising three Spanish populations, two Italian populations from Piedmont and Sicily, two Greek populations, and one Turkish population [24], were combined with the four Lazio stands represented by the conservative post-COLONY dataset. The resulting reference dataset included 12 populations and 309 genotypes.

STRUCTURE analysis identified K = 2 as the strongest broad-scale solution for the five-locus dataset, with K = 3 providing a stable secondary partition (Figure S5; Table S11). These K values are best interpreted as pragmatic summaries of broad genetic affinity within the sampled reference panel, not as definitive reconstructions of European chestnut history.

**Figure 2:**
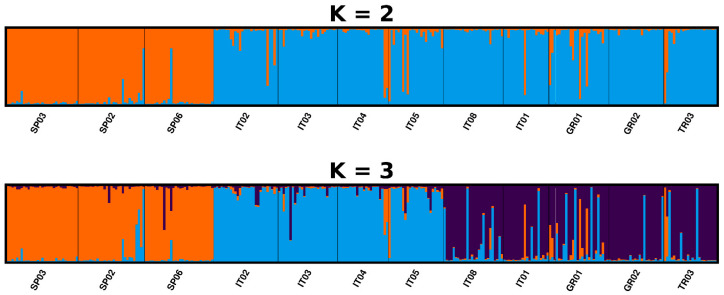
CLUMPAK-aligned ancestry barplots for the 12-population dataset at K = 2 and K = 3. Individuals are ordered by population (SP03, SP02, SP06, IT02, IT03, IT04, IT05, IT08, IT01, GR01, GR02, and TR03). Only the K values retained for interpretation are shown.

At K = 2, the main partition separated the three Spanish populations (SP03, SP02, and SP06) from all remaining populations (Table 6; Figure 2). Spanish populations showed high membership in Cluster 1 (Q = 0.94–0.98), whereas Italian, Greek, and Turkish populations were dominated by Cluster 2, indicating a broad west–east differentiation with a distinct Iberian component.

At K = 3, the Spanish cluster remained stable, while the non-Spanish group split into two components. The Lazio populations (IT02-IT05) were assigned mainly to Cluster 2 (Q = 0.86–0.93), whereas the remaining Italian, Greek, and Turkish populations showed higher membership in Cluster 3. This solution therefore resolved the Lazio stands as a coherent genetic group within the broader non-Spanish background.

The supplementary STRUCTURE summary supports this cautious interpretation (Table S11; Figure S5). Although the Puechmaille estimators suggested additional subdivision, their preferred K values varied across thresholds and tended to favor higher K values. In contrast, Delta K and CLUMPAK consistently supported K = 2 as the main summary and K = 3 as a stable secondary partition. Given the five-locus dataset and uneven sampling, K = 2 and K = 3 were retained as the most robust and interpretable levels of structure.

The K = 2 and K = 3 patterns are consistent with broad phylogeographic expectations for European sweet chestnut, including separation of the Iberian component and differentiation within the non-Iberian background [4,5,6]. However, because this comparison relies on only five shared SSR loci, it should be viewed as evidence of broad genetic affinity rather than a detailed reconstruction of demographic history. Within this framework, the coherent grouping of the Lazio populations is biologically plausible, whereas the more admixed profile of the remaining Italian, Greek, and Turkish populations may reflect historical connectivity, postglacial expansion, and human-mediated movement of chestnut germplasm [62,63].

Genetic diversity was generally high across populations, with Spanish populations often showing the highest values and Lazio populations showing intermediate levels overall (Table S12). Expected heterozygosity ranged from 0.704 to 0.824, allelic richness from 6.667 to 11.013, and private alleles were concentrated mainly in SP03, SP06, IT08, and TR03. The five-locus panel therefore retained enough variation to detect broad-scale structure and population-level diversity differences, but not enough to infer detailed historical processes. The high diversity observed in western populations may reflect multiple postglacial colonization routes, as reported more broadly for trees [72], together with human-mediated dispersal and management.

### 3.6. Development and Optimization of DNA Extraction from Dried Wood and SSR Amplification for Timber Traceability

Extraction of amplifiable DNA from dried chestnut wood required protocol optimization because woody tissues contain limited endogenous DNA and high levels of PCR-inhibiting compounds, as commonly reported for processed or inhibitor-rich plant matrices [73,74]. Preliminary tests with conventional CTAB extraction, phenol–chloroform purification, commercial plant DNA kits, and combined CTAB/silica-column workflows produced low or poorly amplifiable DNA. The final optimized protocol therefore consisted of a modified CTAB extraction followed by PEG purification. The main modifications to the standard CTAB workflow were introduced to improve DNA recovery from degraded and inhibitor-rich material. Prolonged CTAB lysis was used to enhance cell disruption and nucleic acid solubilization [73], while extended isopropanol and sodium acetate precipitation steps were applied to increase DNA recovery [74,75]. The subsequent PEG purification step was included to reduce co-extracted contaminants and recover DNA fractions more suitable for PCR amplification from complex biological matrices [76,77]. Because complete removal of polysaccharides and phenolic compounds is difficult in woody substrates, PCR was further optimized by using HOT FIREPol Taq polymerase together with bovine serum albumin, which can mitigate inhibitory effects on polymerase activity and primer annealing [77,78].

**Figure 3:**
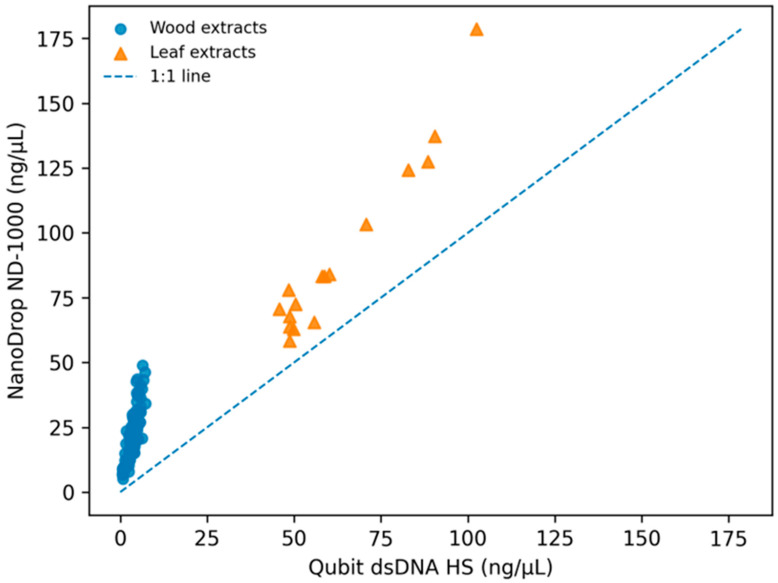
Relationship between NanoDrop and Qubit DNA quantification in wood and leaf extracts. The dashed line represents perfect agreement between the two methods.

DNA quantification confirmed the expected contrast between leaf- and wood-derived extracts. Individual NanoDrop, Qubit, and PCR results are reported in Table S13, and summary statistics are shown in Table S14. Leaf DNA extracted with the NucleoSpin Plant II kit showed higher concentrations and closer agreement between NanoDrop and Qubit estimates. Wood-derived DNA had a median NanoDrop concentration of 20.3 ng/µL and a median Qubit dsDNA concentration of 3.6 ng/µL, with a median NanoDrop/Qubit ratio of 6.38. Paired NanoDrop and Qubit estimates differed significantly within both materials (Wilcoxon signed-rank tests, *p* < 0.001), and Qubit dsDNA concentrations were significantly lower in wood than in leaves (Wilcoxon rank-sum test, *p* < 0.001). The high NanoDrop/Qubit ratio and lower A260/A230 values in wood extracts indicate that spectrophotometric readings likely included non-DNA absorbance from co-extracted compounds [75], emphasizing the need for fluorometric quantification before SSR amplification (Figure 3).

Despite the low Qubit values obtained from wood, the optimized CTAB-PEG workflow yielded SSR-amplifiable DNA from all 40 timber samples. Across independent extractions, 96 of 120 wood-derived DNA extracts amplified successfully for the selected SSR loci, giving an overall success rate of 80.0% (95% CI: 72.0–86.2%). Success rates were similar among timber sources (76.7–83.3%; Table S15), and all 40 samples produced at least two successful amplifications out of three independent extractions, supporting the reproducibility of the workflow at the sample level.

Qubit concentration was the most informative predictor of PCR success (Figure S6). No extract with a Qubit value below 2 ng/µL amplified successfully, whereas 96 of 98 extracts with Qubit values of at least 2 ng/µL were PCR-positive. This association was significant (Fisher’s exact test, *p* < 0.001), and PCR-positive extracts had higher Qubit dsDNA concentrations than failed extracts (Wilcoxon rank-sum test, *p* < 0.001). Qubit quantification therefore provided a simple operational criterion for identifying wood-derived DNA extracts suitable for SSR amplification.

Amplification quality was further supported by agarose gel electrophoresis and capillary electrophoresis. Representative gels obtained with SSR loci CsCAT1 and CsCAT3 from wood samples of different origins and reference leaf DNA confirmed fragments of the expected size (Figure S7). Capillary electrophoresis produced well-resolved allele peaks comparable to those obtained from leaf-derived DNA. In the subset of timber samples subjected to replicate analysis, allele calls from independent extracts were concordant, with no systematic evidence of allelic dropout or peak ambiguity. Extraction blanks and no-template PCR controls did not produce interpretable SSR profiles, supporting the reliability of the workflow and the absence of detectable contamination under the adopted conditions. Overall, the quantitative and amplification data show that the modified CTAB-PEG protocol, combined with PCR optimization, enabled reliable recovery of multilocus SSR genotypes from dried chestnut wood and provided a sound basis for the subsequent assignment analyses.

### 3.7. Reference-Based Assignment of Timber Samples

Reference-based assignment analyses tested whether the 40 timber samples were genetically compatible with the declared Lazio origin or showed stronger affinity for extra-regional references. Reference and timber genotypes were kept analytically separate: reference samples were used to train or estimate the models, whereas timber samples were treated as external unknowns. Supervised DAPC provided posterior membership probabilities, STRUCTURE with USEPOPINFO estimated Q-values relative to predefined reference populations, and GDA_NT 2021 added likelihood-based assignment and exclusion probabilities. Results were interpreted primarily at the individual level and then summarized by sawmill.

#### 3.7.1. Performance of the Reference Panels

The two reference panels showed moderate but useful discriminatory power (Table 7). In the Lazio-only dataset, DAPC correctly classified 76.0% of reference individuals and GDA_NT leave-one-out self-assignment reached 60.0%, indicating an informative but not definitive stand-level signal. In the European/Mediterranean dataset, DAPC correctly classified 74.1% of reference individuals, whereas GDA_NT self-assignment was 60.0% overall and 58.0% as the mean across populations. These values support regional consistency testing, but they also show that individual stand-level assignment should be interpreted cautiously, particularly among genetically close Lazio populations and in the presence of geographically incomplete reference panels. Detailed reference-panel outputs and individual assignment results are reported in Tables S16–S18.

#### 3.7.2. Assignment Within the Lazio Reference Panel Using Seven SSR Loci

In the Lazio-only dataset, DAPC assigned MCt mainly to IT02 and RPt mainly to IT03, consistent with the declared origin of the two Lazio sawmills (Table 8; Figure S8; Table S16). Posterior probabilities were not fully exclusive, reflecting weak differentiation among Lazio stands. Because this dataset included only Lazio reference populations, CALt and FRAt were necessarily assigned to the closest available Lazio group by DAPC; these relative assignments should therefore not be interpreted as evidence of a Lazio origin.

STRUCTURE was more conservative. The highest mean Q-values tended toward IT02 for MCt and toward IT03/IT04 for RPt, but maximum mean Q-values were low (approximately 0.27–0.28) and broadly distributed among the four Lazio populations (Table S17). Thus, STRUCTURE indicated weak affinity patterns rather than robust stand-level assignment. GDA_NT provided the strongest compatibility test in this restricted panel: MCt and RPt were compatible with Lazio references, whereas CALt and FRAt showed high exclusion probabilities (Table S18). The supplementary sawmill-level GDA_NT analysis confirmed the same qualitative pattern and is reported only as a consistency check (Table S19; Figure S9).

#### 3.7.3. Assignment Within the European/Mediterranean Reference Panel Using Five SSR Loci

**Figure 4:**
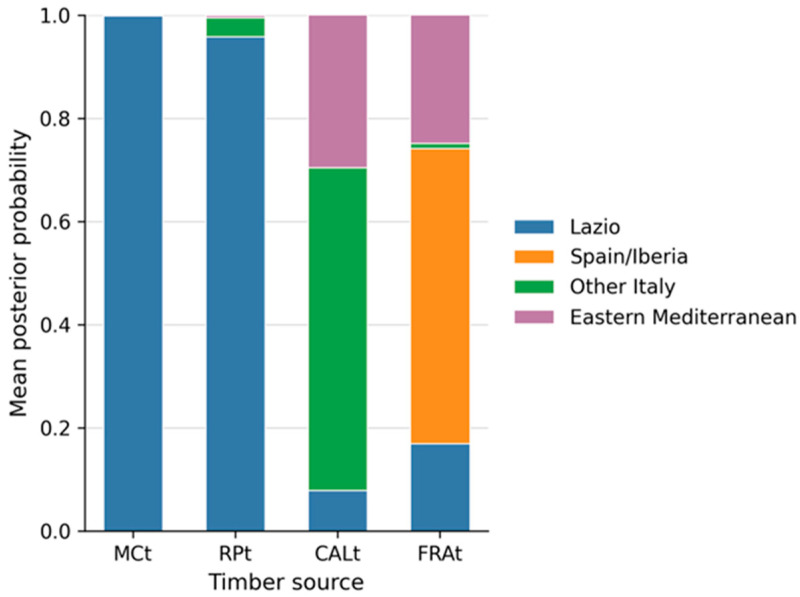
Mean DAPC posterior probabilities of the four timber sources in the European/Mediterranean assignment dataset, aggregated by macro-region.

**Figure 5:**
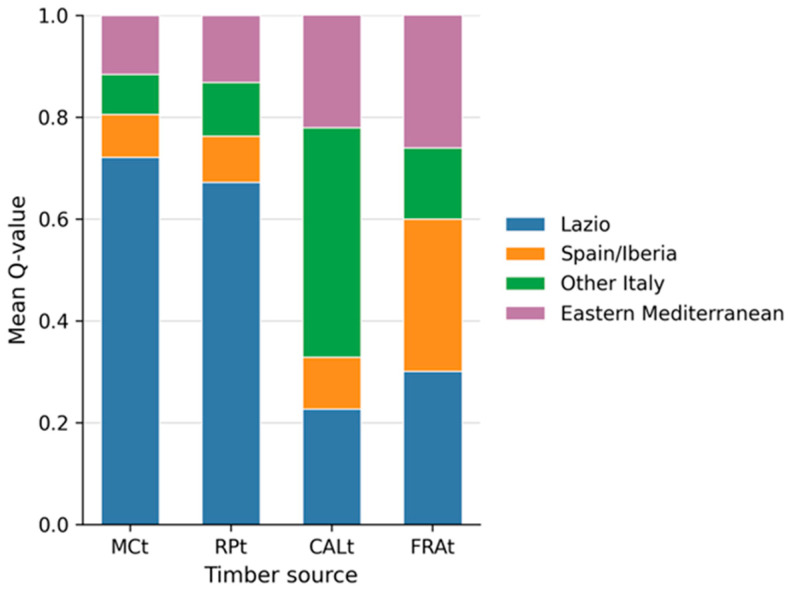
Mean STRUCTURE Q-values of the four timber sources in the European/Mediterranean assignment dataset, aggregated by macro-region.

The European/Mediterranean reference panel clarified the regional interpretation of the timber samples (Table 9; Figure 4 and Figure 5; Figure S10). DAPC assigned all MCt and RPt individuals to Lazio populations, with very high combined posterior probabilities for the Lazio group (0.999 and 0.958, respectively; Table S16). STRUCTURE gave the same regional signal, with the highest aggregate Q-values for Lazio in MCt (0.721) and RPt (0.672; Table S17). GDA_NT also assigned all MCt and RPt individuals to Lazio populations, mainly IT02 and IT03, without exclusion at the 95% threshold (Table S18). These results support the declared Lazio origin of both sawmill groups, although resolution among individual Lazio stands remained moderate.

CALt and FRAt showed extra-regional patterns. CALt was assigned mainly to non-Lazio Italian populations, especially IT01 and IT08, with some affinity for GR01; STRUCTURE also showed a predominant Other Italy component. This pattern indicates extra-Lazio affinity and is consistent with the absence of a specific Calabria reference population. FRAt was less resolved: DAPC indicated the strongest affinity with the Iberian group, especially SP02, whereas STRUCTURE showed a mixed profile with similar Lazio, Iberian, and eastern Mediterranean components. GDA_NT assigned several FRAt individuals to SP02 or other Mediterranean references, but almost all were excluded at the 95% threshold. FRAt should therefore be interpreted as extra-Lazio and incompletely represented by the current reference panel, rather than confidently assigned to a sampled population.

The macro-regional signal is summarized in Figure 4 and Figure 5. DAPC showed almost complete Lazio affinity for MCt and RPt, non-Lazio Italian affinity for CALt, and Iberian affinity for FRAt. STRUCTURE recovered the same broad pattern for MCt, RPt and CALt, but returned a more diffuse profile for FRAt. Individual posterior probabilities, Q-values and GDA_NT exclusion results are provided in Tables S16–S18, while the supplementary GDA_NT sawmill-level consistency check is reported in Table S19 and Figure S9. Together, these supplementary outputs document how the individual-level evidence supports the integrated interpretation.

#### 3.7.4. Concordance Among DAPC, STRUCTURE, and GDA_NT

The three methods were broadly concordant at the regional scale. DAPC provided the clearest separation among predefined reference groups, but its assignments remain conditional on reference-panel coverage. STRUCTURE with USEPOPINFO was more conservative and highlighted uncertainty when differentiation among candidate populations was weak. GDA_NT added formal exclusion testing, distinguishing the closest relative match from statistical compatibility with a reference population. The strongest conclusion is therefore regional: MCt and RPt are compatible with the Lazio gene pool, CALt is inconsistent with Lazio and shows non-Lazio Italian affinity, and FRAt is not reliably represented by the current reference panel despite partial Iberian affinity. Detailed individual-level evidence is provided in Tables S16–S18 and Figures S8–S10.

#### 3.7.5. Implications and Limitations for Timber Traceability

Taken together, DAPC, STRUCTURE and GDA_NT indicate that SSR profiles obtained from wood-derived DNA can support regional provenance assessment in *C. sativa*, while also highlighting the limited resolution of stand-level assignment in this dataset. MCt and RPt were consistently compatible with the Lazio gene pool and with the declared origin of the two Lazio sawmills, whereas CALt and FRAt were not supported as Lazio material. The current reference panel is geographically limited and should not be considered representative of the full genetic diversity of *C. sativa* across Italy or Europe. Assignment results are therefore best interpreted as tests of compatibility or incompatibility with the sampled reference populations, rather than as the definitive identification of precise geographic origin.

These results are relevant to timber traceability under regulatory frameworks requiring verification of timber origin, including European due-diligence requirements [22,23]. They are consistent with forensic timber identification guidelines emphasizing validated methods, reference data and cautious communication of evidence [79], and with recent reviews showing that geographic origin testing strongly depends on representative reference datasets [15,16]. Accordingly, the present results should be regarded as a proof of concept for regional chestnut provenance screening rather than as a stand-level forensic tool.

SSR markers remain operationally accessible and informative for distinguishing major gene pools in European chestnut when supported by appropriate reference panels [5,6,9,24]. In this study, they provided a practical compromise between resolution, feasibility, and cost because SSR genotyping can be implemented with relatively simple workflows and lower routine costs than high-density SNP approaches. Nevertheless, moderate self-assignment rates, weak differentiation among Lazio stands and the absence of French and Calabrian reference populations limit fine-scale inference. Higher-density nuclear and organellar SNP panels may improve assignment resolution when combined with extensive baselines [80], but they should be considered a complementary future development rather than a prerequisite for all applications. Until broader reference resources are available, SSR-based assignment can serve as an independent biological line of evidence for regional screening and provenance verification, provided that results are interpreted conservatively and supported by appropriate reference data.

## 4. Conclusions

This study integrated population genetic analysis, wood-derived DNA recovery, and reference-based timber assignment to assess genetic diversity and provenance verification in Lazio coppice chestnut forests. The four Lazio populations retained high multilocus SSR diversity, comparable to that of other European chestnut resources, but also showed widespread heterozygote deficiency, positive inbreeding coefficients, and fine-scale family structure. The conservative post-COLONY dataset therefore represented a more robust basis for downstream population structure and assignment analyses while preserving sufficient genetic variation.

Population structure analyses indicated weak but detectable differentiation among Lazio stands, with most genetic variation distributed within populations. This pattern is consistent with a connected regional gene pool shaped by the outcrossing biology of sweet chestnut, long-term coppice management, and gene flow among neighboring stands. In the available European and Mediterranean reference set, the Lazio populations showed a coherent Central Italian affinity. This broad affinity should be interpreted cautiously because the comparison was based on five shared SSR loci. Even so, the results provide useful baseline information for conserving and managing local coppice resources by maintaining reproductive heterogeneity, local diversity, and connectivity among stands.

The study also established an effective workflow for obtaining SSR-amplifiable DNA from dried chestnut wood. The optimized CTAB-PEG protocol, combined with PCR adjustments for inhibitor-rich material, produced reliable multilocus profiles from all timber samples retained for assignment. Independent extractions, negative controls, and replicate checks supported the reproducibility of wood-derived genotypes and reduced the likelihood that assignment patterns reflected contamination or scoring artifacts. This technical step is essential for translating population genetic data into operational provenance testing of timber material.

Reference-based assignment analyses provided a cautious but informative assessment of timber origin. Across supervised DAPC, STRUCTURE with population information, and GDA_NT Bayesian assignment and exclusion testing, the two Lazio timber groups were consistently compatible with the Lazio gene pool and with their declared local origin. Conversely, the Calabria and French timber groups were not supported as Lazio material. The Calabria samples showed stronger extra-Lazio, mainly non-Lazio Italian, affinity, whereas the French samples were less clearly resolved and likely not fully represented by the available reference panel. These results support the use of SSR profiles for regional provenance assessment while confirming that assignment to individual stands remains limited when reference populations are weakly differentiated and reference coverage is incomplete.

Overall, SSR markers, when combined with carefully selected reference datasets and formal assignment/exclusion procedures, provide a practical first-line tool for regional chestnut timber provenance verification. Their relative simplicity, established laboratory workflow, and lower routine cost make them useful for preliminary screening and for supporting local traceability systems. However, SSR-based assignment should not be considered a stand-alone legal proof of origin. Broader georeferenced reference panels, particularly from Southern Italy, France, and the western Mediterranean, together with complementary high-resolution nuclear and organellar markers, would improve future assignment resolution. Within these limits, the integrated framework developed here provides both a scientific basis for the conservation and management of Lazio coppice chestnut forests and a preliminary operational model for regional chestnut timber traceability.

## Figures and Tables

**Table 1:** Location, code, and number of individuals (N) of coppice chestnut populations from four selected areas of the Lazio Region, and eight European reference populations.

Country	Region	Population	Code	N	Reference
Italy	Lazio	San Martino al Cimino	IT02	40	This study
Italy	Lazio	Rocca di Papa	IT03	40	This study
Italy	Lazio	Oriolo Romano	IT04	40	This study
Italy	Lazio	Fiuggi	IT05	40	This study
Italy	Piedmont	Villar Pellice	IT08	26	Castellana et al. [24]
Italy	Sicily	Madonie	IT01	20	Castellana et al. [24]
Spain	Galicia	Costa Atlantica	SP03	31	Castellana et al. [24]
Spain	Catalonia	Castanyet	SP02	29	Castellana et al. [24]
Spain	Extremadura	Hervas	SP06	30	Castellana et al. [24]
Greece	S-E Macedonia	Holomontas	GR01	26	Castellana et al. [24]
Greece	C-Macedonia	Hortiatis	GR02	24	Castellana et al. [24]
Turkey	Black Sea	Hopa	TR03	23	Castellana et al. [24]

**Table 2:** Genetic diversity parameters of the 12 SSR loci analyzed in 160 trees from chestnut coppice stands in the Lazio region, including number of alleles (Na), effective number of alleles (Ne), major allele frequency (MAF), Shannon’s information index (I), observed heterozygosity (Ho), expected heterozygosity (He), unbiased expected heterozygosity (uHe), inbreeding coefficient (F), and polymorphic information content (PIC).

Locus	Na	Ne	MAF	I	Ho	He	uHe	F	PIC
CsCAT1	7.00	3.444	0.484	1.499	0.669	0.696	0.705	0.036	0.686
CsCAT14	6.00	2.908	0.475	1.224	0.650	0.652	0.660	0.003	0.613
CsCAT3	14.00	5.085	0.316	1.853	0.525	0.795	0.805	0.336	0.794
CsCAT16	11.00	3.516	0.425	1.473	0.619	0.704	0.713	0.107	0.688
CsCAT2	10.00	5.819	0.203	1.896	0.456	0.827	0.837	0.448	0.833
CsCAT6	11.00	4.732	0.328	1.769	0.631	0.782	0.792	0.191	0.777
CsCAT34	7.00	3.682	0.303	1.408	0.400	0.715	0.724	0.421	0.722
CsCAT17	11.00	5.384	0.212	1.850	0.794	0.814	0.824	0.025	0.849
CsCAT41	7.00	4.883	0.244	1.695	0.444	0.795	0.805	0.441	0.792
EMCs32	7.00	2.984	0.469	1.287	0.250	0.662	0.670	0.631	0.650
EMCs25	6.00	2.346	0.547	1.102	0.213	0.568	0.575	0.612	0.567
EMCs38	13.00	6.830	0.237	2.132	0.538	0.852	0.862	0.370	0.874
Mean	9.17	4.301	0.354	1.599	0.516	0.738	0.748	0.302	0.737
Total	110.00								

**Table 3:** Summary comparison of locus-based diversity statistics in the original, conservative, and severe datasets after COLONY-based filtering.

Dataset	N Individuals	Total Alleles	Mean Na	Mean Ne	Mean Ho	Mean He	Mean F	Mean PIC
Original	160	110	9.17	4.900	0.516	0.768	0.331	0.737
Conservative	100	106	8.83	4.941	0.526	0.770	0.316	0.742
Severe	73	104	8.67	5.029	0.524	0.775	0.322	0.747

**Table 4:** Population structure analysis for the conservative dataset at K = 4. Mean membership proportions (Q) to the four inferred clusters are shown for each Lazio population; the main contribution in each population is shown in bold.

Population	N	Cluster 1	Cluster 2	Cluster 3	Cluster 4
IT02	28	0.186	0.277	0.229	**0.308**
IT03	26	0.161	0.281	**0.292**	0.266
IT04	22	0.232	**0.311**	0.152	0.304
IT05	24	**0.354**	0.180	0.304	0.163

**Table 5:** AMOVA-style variance partitioning, fixation index (Fst), standardized G’st, and gene flow (Nm) for the four Lazio chestnut populations based on the conservative dataset. Significance of Fst was assessed with 999 permutations. *** indicates significance at *p* < 0.001.

Source	df	SS	MS	Est. Var.	%	Fst	G’st	Nm
Among populations	3	78.888	26.296	0.346	3.7%			
Within populations	196	1769.772	9.029	9.029	96.3%			
Total	199	1848.660		9.376	100%	0.037 ***	0.219	6.519

**Table 6:** Mean membership proportions (Q) of the 12 populations at K = 2 and K = 3 based on the CLUMPAK-aligned major mode. The dominant cluster at each K is shown in bold.

	K = 2		K = 3		
Population	Cluster 1	Cluster 2	Cluster 1	Cluster 2	Cluster 3
SP03	**0.9767**	0.0233	**0.9589**	0.0218	0.0193
SP02	**0.9366**	0.0634	**0.8702**	0.1055	0.0242
SP06	**0.9570**	0.0431	**0.9312**	0.0217	0.0471
IT02	0.0814	**0.9186**	0.0245	**0.9344**	0.0411
IT03	0.0391	**0.9609**	0.0224	**0.9104**	0.0671
IT04	0.0961	**0.9039**	0.0582	**0.9096**	0.0322
IT05	0.1655	**0.8345**	0.0854	**0.8582**	0.0564
IT08	0.0271	**0.9729**	0.0251	0.1722	**0.8027**
IT01	0.0748	**0.9252**	0.0544	0.0896	**0.8560**
GR01	0.1646	**0.8354**	0.1214	0.1913	**0.6872**
GR02	0.0186	**0.9814**	0.0104	0.0909	**0.8987**
TR03	0.0916	**0.9084**	0.0764	0.1178	**0.8058**

**Table 7:** Reference-panel performance across the two assignment datasets. DAPC performance was based on the reference-sample confusion matrix, whereas GDA_NT performance was assessed using leave-one-out self-assignment with the group size set to 1.

Dataset	Reference Panel	Loci	Method	Reference-Panel Performance	Interpretation
Lazio	IT02-IT05, conservative post-COLONY dataset (N = 100)	7	DAPC	76.0% correct reference classification	Moderate stand-level discrimination
Lazio	IT02-IT05, conservative post-COLONY dataset (N = 100)	7	GDA_NT	60.0% leave-one-out self-assignment	Moderate stand-level discrimination
European/ Med.	12 populations, including Lazio conservative dataset (N = 309)	5	DAPC	74.1% correct reference classification	Useful macro-regional discrimination
European/ Med.	12 populations, including Lazio conservative dataset (N = 309)	5	GDA_NT	60.0% overall; 58.0% mean across populations	Variable performance; strongest for Spanish populations

**Table 8:** Integrated assignment summary for the Lazio-only seven-locus dataset. GDA_NT compatibility indicates that the assigned reference population was not excluded at the 95% threshold. For CALt and FRAt, the Lazio-only reference panel tests compatibility with Lazio populations only and cannot identify the true extra-regional source.

Timber Source	Declared Origin	DAPC Individual Assignments	STRUCTURE Mean Q Summary	GDA_NT Individual Assignment/Exclusion	Integrated Interpretation
MCt	Lazio, IT02/Cimini	IT02 = 5; IT03 = 2; IT04 = 1; IT05 = 2	Highest mean Q: IT02 (0.281); mean maxQ = 0.284	Cimini_POP_1 = 7; OrioloR_POP_3 = 2; RPapa_POP_2 = 1; compatible = 10/10	Compatible with Lazio; strongest support toward IT02 but stand-level resolution remains moderate.
RPt	Lazio, IT03/RPapa	IT03 = 8; IT04 = 1; IT05 = 1	Highest mean Q: IT03 (0.269); mean maxQ = 0.272	RPapa_POP_2 = 10; compatible = 10/10	Compatible with Lazio; strongest support toward IT03/Rocca di Papa.
CALt	Calabria, extra-Lazio	IT03 = 2; IT04 = 1; IT05 = 7	Highest mean Q: IT03 (0.277); mean maxQ = 0.283	RPapa_POP_2 = 5; Fiuggi_POP_4 = 4; OrioloR_POP_3 = 1; compatible = 0/10	Not compatible with Lazio references; Lazio-only DAPC/STRUCTURE assignment is forced.
FRAt	France, extra-Lazio	IT02 = 1; IT03 = 1; IT04 = 3; IT05 = 5	Highest mean Q: IT03 (0.271); mean maxQ = 0.279	Fiuggi_POP_4 = 4; Cimini/RPapa/Oriolo = 2 each; compatible = 0/10	Not compatible with Lazio references; true origin absent from this restricted panel.

**Table 9:** Integrated assignment summary for the European/Mediterranean five-locus dataset. Macro-regions were defined as Lazio (IT02-IT05), Spain/Iberia (SP02, SP03, and SP06), Other Italy (IT01 and IT08), and Eastern Mediterranean (East. Medit.) (GR01, GR02, and TR03).

Timber Source	Declared Origin	DAPC Mean Posterior by Macro-Region	STRUCTURE Mean Q by Macro-Region	GDA_NT Individual Assignment/Exclusion	Integrated Interpretation
MCt	Lazio, IT02/Cimini	Lazio = 0.999; Spain/Iberia = 0.000; Other Italy = 0.001; East. Medit. = 0.000	Lazio = 0.721; Spain/Iberia = 0.084; Other Italy = 0.079; East. Medit. = 0.116	IT02 = 6; IT03 = 1; IT04 = 2; IT05 = 1; Compatible = 10/10	Strong regional support for Lazio, with prevailing IT02 affinity.
RPt	Lazio, IT03/RPapa	Lazio = 0.958; Spain/Iberia = 0.000; Other Italy = 0.037; East. Medit. = 0.005	Lazio = 0.672; Spain/Iberia = 0.091; Other Italy = 0.106; East. Medit. = 0.132	IT03 = 7; IT04 = 3; Compatible = 10/10	Strong regional support for Lazio, with prevailing IT03/IT04 affinity.
CALt	Calabria, extra-Lazio	Lazio = 0.079; Spain/Iberia = 0.001; Other Italy = 0.625; East. Medit. = 0.296	Lazio = 0.227; Spain/Iberia = 0.102; Other Italy = 0.451; East. Medit. = 0.221	IT01 = 4; IT08 = 3; GR01 = 2; IT04 = 1; Compatible = 8/10; Lazio assignment excluded	Extra-Lazio; closest affinity to non-Lazio Italian references, not Lazio.
FRAt	France, extra-Lazio	Lazio = 0.170; Spain/Iberia = 0.572; Other Italy = 0.011; East. Medit. = 0.248	Lazio = 0.301; Spain/Iberia = 0.299; Other Italy = 0.140; East. Medit. = 0.261	SP02 = 3; GR01 = 2; IT02 = 2; IT03/IT04/IT05 = 1 each; compatible = 1/10	Extra-Lazio; relative Iberian affinity but true source likely absent from reference panel.

## Data Availability

Data supporting the findings of this study are provided in the article and Supplementary Materials. Additional information is available from the corresponding author upon reasonable request.

## Supplementary Materials

The following supporting information can be downloaded at: https://www.mdpi.com/article/10.3390/plants15132066/s1. Figure S1. Map showing the four locations of coppice chestnut forests in the selected areas of the Lazio region considered in this study. Figure S2. Mean LnP(K) and Evanno ΔK across the original, conservative, and severe post-COLONY datasets. The original dataset shows the strongest ΔK peak at K = 2, the conservative dataset peaks at K = 4, and the severe dataset shows the weakest overall signal. Figure S3. CLUMPAK/DISTRUCT ancestry barplots at K = 5 for the original, conservative, and severe datasets. Panels were assembled from the original CLUMPAK/DISTRUCT export files for the major mode after CLUMPP alignment. Relative to K = 4, this solution introduces finer subdivision but does not improve biological interpretability or assignment sharpness. Figure S4. Custom heat map summarizing Puechmaille-selected K values across thresholds (0.5–0.8) for the original, conservative, and severe datasets, based on STRUCTURE SELECTOR output tables. Cell values indicate the K selected by each estimator (MedMedK, MedMeanK, MaxMedK, and MaxMeanK) at each threshold. Figure S5. STRUCTURE SELECTOR summary for the 12-population dataset. Panel A shows mean LnP(K) across K = 1–12; panel B shows the Evanno Delta K statistic. The strongest support occurred at K = 2, with a secondary peak at K = 3. Figure S6. SSR amplification success across Qubit dsDNA concentration classes in wood extracts. Numbers above bars indicate successful amplifications/total extracts within each class. Figure S7. Agarose gel (1.2%) electrophoresis of PCR amplification products obtained from DNA extracted from woody tissue of different origins (samples 1–10) and from leaf tissue (sample 11) used as a control. Lanes 1–3: Lazio, Rocca di Papa; lanes 4–5: Calabria; lanes 6–7: France; lanes 8–10: Lazio, San Martino al Cimino; lane 11: control leaf sample; M: molecular marker (100 bp DNA ladder). Figure S8. Supervised DAPC of the Lazio seven-locus assignment dataset. Reference individuals were used to fit the DAPC model, whereas timber samples were projected a posteriori. Figure S9. GDA_NT exclusion probabilities from the supplementary sawmill-level assignment analyses. The dashed horizontal line indicates the 95% exclusion threshold. D1 = Dataset 1; D2 = Dataset 2. Figure S10. Supervised DAPC of the European/Mediterranean five-locus assignment dataset. Reference individuals were used to fit the DAPC model, whereas timber samples were projected a posteriori. Table S1. Characteristics of the 12 SSR loci used in this study. Table S2. Private SSR alleles detected in four Lazio chestnut coppice stands in homozygous (Hom) and heterozygous (Het) states. Numbers in brackets indicate the individuals carrying each allele. Table S3. Population-level summary of HWE-related statistics based on GenAlEx and Genepop. Table S4. Integrated locus-level summary from GenAlEx-style statistics and Genepop exact tests. Table S5. INEst2 summary by population. Potential null-allele candidates are reported from the nf model for conservative cross-population comparison. Table S6. COLONY summary of within-population family structure and reduced datasets for downstream sensitivity analyses. Table S7. Comparative summary of STRUCTURE SELECTOR and CLUMPAK evidence used to evaluate the three post-COLONY datasets. Table S8. Comparison between K = 4 and K = 5 in terms of CLUMPAK mode stability and assignment sharpness. Table S9. Pairwise differentiation among the four coppice chestnut populations from Lazio based on Nei’s unbiased genetic distance (Nei uD), Fst, standardized G’st, and Nm for the conservative dataset. Table S10. Genetic diversity of the four Lazio chestnut populations in the conservative dataset. Npa = number of private alleles; Na = mean number of alleles per locus; Ne = mean effective number of alleles; I = Shannon’s information index; Ho = observed heterozygosity; He = expected heterozygosity; uHe = unbiased expected heterozygosity; F = inbreeding coefficient. Table S11. Summary of STRUCTURE and CLUMPAK metrics for the 12 population dataset. Panel A reports K-specific LnP(K), ΔK, and major-mode stability. Panel B reports the Puechmaille estimators across thresholds. Panel A. K-specific model-selection and stability metrics. Panel B. Puechmaille estimator summaries across thresholds. Table S12. Genetic diversity parameters of the four Lazio coppice chestnut populations and the eight European populations used for comparison, calculated from five SSR loci. NI = number of individuals; Npa = number of private alleles; Na = number of alleles per locus; Ne = effective number of alleles; I = Shannon’s information index; Ho = observed heterozygosity; He = expected heterozygosity; uHe = unbiased expected heterozygosity; F = inbreeding coefficient; Ar = allelic richness. Table S13. Individual NanoDrop, Qubit, and PCR results for 120 independent timber DNA extractions and 16 leaf DNA reference controls (Table S13 has been provided as a separate spreadsheet). Table S14. Summary of DNA quantity and quality in wood and leaf extracts. Values are reported as median (interquartile range). Table S15. SSR amplification success in independent wood DNA extractions. Each timber source included 10 timber samples and three independent extractions per sample. Successful extracts indicate independent DNA extractions that generated scorable SSR-PCR profiles. Table S16. DAPC reference-sample confusion matrices and individual timber assignments for the Lazio and European/Mediterranean datasets. Full individual posterior probabilities are available as a separate spreadsheet. D1 = Dataset 1; D2 = Dataset 2. Table S17. STRUCTURE individual Q-values and sawmill-level summaries for the two reference-based assignment datasets. Q-values were averaged across 20 independent runs before summarization. D1 = Dataset 1; D2 = Dataset 2. Table S18. GDA_NT individual assignment results for each timber sample, including -log10 genotype likelihood, assignment score, and exclusion probability. Compatible indicates that the assigned reference population was not excluded at the 95% threshold. D1 = Dataset 1; D2 = Dataset 2. Table S19. Supplementary GDA_NT sawmill-level group assignment for the two assignment datasets. This group-level analysis was used only as a consistency check and was not considered the primary measure of traceability performance. D1 = Dataset 1; D2 = Dataset 2.

## References

[1] Conedera M., Tinner W., Krebs P., de Rigo D., Caudullo G., San-Miguel-Ayanz J., de Rigo D., Caudullo G., Houston Durrant T., Mauri A. (2016). *Castanea sativa* in Europe: Distribution, habitat, usage and threats. European Atlas of Forest Tree Species.

[2] Food and Agriculture Organization of the United Nations (FAO) (2020). Global Forest Resources Assessment 2020-Main Report.

[3] Conedera M., Krebs P., Tinner W., Pradella M., Torriani D. (2004). The cultivation of *Castanea sativa* (Mill.) in Europe: From its origin to its diffusion on a continental scale. Veget. Hist. Archaeobot..

[4] Krebs P., Conedera M., Pradella M., Torriani D., Felber M., Tinner W. (2004). Quaternary refugia of the sweet chestnut (*Castanea sativa* Mill.): An extended palynological approach. Veget. Hist. Archaeobot..

[5] Mattioni C., Màrtin M.A., Pollegioni P., Cherubini M., Villani F. (2013). Microsatellite markers reveal a strong geographical structure in European populations of *Castanea sativa* (Fagaceae): Evidence for multiple glacial refugia. Am. J. Bot..

[6] Mattioni C., Màrtin M.A., Chiocchini F., Cherubini M., Gaudet M., Pollegioni P., Velichkov I., Jarman R., Chambers F.M., Paule L. (2017). Landscape genetics structure of European sweet chestnut (*Castanea sativa* Mill.): Indications for conservation priorities. Tree Genet. Genomes.

[7] Pereira-Lorenzo S., Ramos-Cabrer A.M., Barreneche T., Mattioni C., Villani F., Díaz-Hernández M.B. (2017). Database of European chestnut cultivars and definition of a core collection using simple sequence repeats. Tree Genet. Genomes.

[8] Jarman R., Mattioni C., Russell K., Chambers F.M., Bartlett D., Màrtin M.A., Cherubini M., Villani F., Webb J. (2019). DNA analysis of *Castanea sativa* (sweet chestnut) in Britain and Ireland: Elucidating European origins and genepool diversity. PLoS ONE.

[9] Bouffartigue C., Debille S., Fabreguettes O., Ramos Cabrer A.M., Pereira-Lorenzo S., Flutre T., Harvengt L. (2020). Two main genetic clusters with high admixture between forest and cultivated chestnut (*Castanea sativa* Mill.) in France. Ann. For. Sci..

[10] Gasparini P., Di Cosmo L., Floris A., De Laurentis D. (2022). Italian National Forest Inventory-Methods and Results of the Third Survey. Springer Tracts in Civil Engineering.

[11] Caré O., Kuchma O., Hosius B., Voth W., Thurm E.A., Leinemann L. (2024). Patterns of genetic variation and the potential origin of sweet chestnut (*Castanea sativa* Mill.) stands far from its natural northern distribution edge. Silvae Genet..

[12] Allendorf F.W., Luikart G.H., Aitken S.N. (2012). Conservation and the Genetics of Populations.

[13] Lowe A.J., Cross H.B. (2011). The application of DNA methods to timber tracking and origin verification. IAWA J..

[14] Dormontt E.E., Boner M., Braun B., Breulmann G., Degen B., Espinoza E., Gardner S., Guillery P., Hermanson J.C., Koch G. (2015). Forensic timber identification: It’s time to integrate disciplines to combat illegal logging. Biol. Conserv..

[15] Low M.C., Schmitz N., Boeschoten L., Cabezas J.A., Cramm M., Haag V., Koch G., Meyer-Sand B.R.V., Paredes-Villanueva K., Price E. (2023). Tracing the world’s timber: The status of scientific verification technologies for species and origin identification. IAWA J..

[16] Tonouéwa J.F.M.F., Biaou S.S.H., Assèdé E.S.P., Agossou H., Balagueman R.O. (2024). Timber traceability, determining effective methods to combat illegal logging in Africa: A review. Trees For. People.

[17] Gasson P.E., Lancaster C.A., Young R., Redstone S., Miles-Bunch I.A., Rees G., Guillery R.P., Parker-Forney M., Lebow E.T. (2021). WorldForestID: Addressing the need for standardized wood reference collections to support authentication analysis technologies; a way forward for checking the origin and identity of traded timber. Plants People Planet.

[18] Degen B., Ward S.E., Lemes M.R., Navarro C., Cavers S., Sebbenn A.M. (2013). Verifying the geographic origin of mahogany (*Swietenia macrophylla* King) with DNA fingerprints. Forensic Sci. Int. Genet..

[19] Vlam M., de Groot G.A., Boom A., Copini P., Laros I., Veldhuijzen K., Zakamdi D., Zuidema P.A. (2018). Developing forensic tools for an African timber: Regional origin is revealed by genetic characteristics, but not by isotopic signature. Biol. Conserv..

[20] Deguilloux M.F., Pemonge M.H., Petit R.J. (2002). Novel perspectives in wood certification and forensics: Dry wood as a source of DNA. Proc. R. Soc. B Biol. Sci..

[21] Fatima T., Srivastava A., Hanur V.S., Srinivasa Rao M. (2018). An effective wood-derived DNA extraction protocol for three economically important timber species of India. Am. J. Plant Sci..

[22] European Parliament and Council (2010). Regulation (EU) No. 995/2010 of the European Parliament and of the Council of 20 October 2010 laying down the obligations of operators who place timber and timber products on the market (EU Timber Regulation). Off. J. Eur. Union.

[23] European Parliament and Council (2023). Regulation (EU) 2023/1115 of the European Parliament and of the Council of 31 May 2023 on the making available on the Union market and export from the Union of certain commodities and products associated with deforestation and forest degradation (EU Deforestation Regulation). Off. J. Eur. Union.

[24] Castellana S., Màrtin M.A., Solla A., Alcaide F., Villani F., Cherubini M., Neale D., Mattioni C. (2021). Signatures of local adaptation to climate in natural populations of sweet chestnut (*Castanea sativa* Mill.) from southern Europe. Ann. For. Sci..

[25] Marini F., Portoghesi L., Manetti M.C., Salvati L., Romagnoli M. (2021). Gaps and Perspectives for the Improvement of the Sweet Chestnut Forest-Wood Chain in Italy. Ann. Silvic. Res..

[26] Allevato E., Vinciguerra V., Stazi S.R., Carbone F., Zuccaccia C., Nano G., Marabottini R. (2022). Fulvic Acid from Chestnut Forest as an Added Qualities to Spring Water: Isolation and Characterization from Fiuggi Waters. Minerals.

[27] Doyle J.J., Doyle J.L. (1987). A rapid DNA isolation procedure for small quantities of fresh leaf tissue. Phytochem. Bull..

[28] Buck E.J., Hadonou M., James C.J., Blakesley D., Russell K. (2003). Isolation and characterization of polymorphic microsatellites in European chestnut (*Castanea sativa* Mill.). Mol. Ecol. Notes.

[29] Marinoni D., Akkak A., Bounous G., Edwards K.J., Botta R. (2003). Development and characterization of microsatellite markers in *Castanea sativa* (Mill.). Mol. Breed..

[30] Liu K., Muse S.V. (2005). PowerMarker: An integrated analysis environment for genetic marker analysis. Bioinformatics.

[31] Peakall R., Smouse P.E. (2012). GenAlEx 6.5: Genetic analysis in Excel. Population genetic software for teaching and research—An update. Bioinformatics.

[32] Weber J.L. (1990). Informativeness of human (dC–dA)n·(dG–dT)n polymorphisms. Genomics.

[33] Rousset F. (2008). genepop’007: A Complete Re-Implementation of the genepop Software for Windows and Linux. Mol. Ecol. Resour..

[34] Chybicki I.J., Burczyk J. (2009). Simultaneous Estimation of Null Alleles and Inbreeding Coefficients. J. Hered..

[35] Jones O.R., Wang J. (2010). COLONY: A Program for Parentage and Sibship Inference from Multilocus Genotype Data. Mol. Ecol. Resour..

[36] Pritchard J.K., Stephens M., Donnelly P. (2000). Inference of population structure using multilocus genotype data. Genetics.

[37] Li Y.L., Liu J.X. (2018). StructureSelector: A web-based software to select and visualize the optimal number of clusters using multiple methods. Mol. Ecol. Resour..

[38] Evanno G., Regnaut S., Goudet J. (2005). Detecting the Number of Clusters of Individuals Using the Software STRUCTURE: A Simulation Study. Mol. Ecol..

[39] Puechmaille S.J. (2016). The program STRUCTURE does not reliably recover the correct population structure when sampling is uneven: Subsampling and new estimators alleviate the problem. Mol. Ecol. Resour..

[40] Kopelman N.M., Mayzel J., Jakobsson M., Rosenberg N.A., Mayrose I. (2015). CLUMPAK: A program for identifying clustering modes and packaging population structure inferences across K. Mol. Ecol. Resour..

[41] Hedrick P.W. (2005). A standardized genetic differentiation measure. Evolution.

[42] Nei M. (1978). Estimation of average heterozygosity and genetic distance from a small number of individuals. Genetics.

[43] Goudet J. (2002). FSTAT (Version 2.9.3.2): A Program to Estimate and Test Gene Diversities and Fixation Indices.

[44] Jombart T. (2008). adegenet: A R package for the multivariate analysis of genetic markers. Bioinformatics.

[45] Jombart T., Devillard S., Balloux F. (2010). Discriminant analysis of principal components: A new method for the analysis of genetically structured populations. BMC Genet..

[46] Jombart T., Ahmed I. (2011). adegenet 1.3–1: New tools for the analysis of genome-wide SNP data. Bioinformatics.

[47] Falush D., Stephens M., Pritchard J.K. (2003). Inference of Population Structure Using Multilocus Genotype Data: Linked Loci and Correlated Allele Frequencies. Genetics.

[48] Degen B. (2022). GDA-NT 2021: A Computer Program for Population Genetic Data Analysis and Assignment. Conserv. Genet. Resour..

[49] Rannala B., Mountain J.L. (1997). Detecting Immigration by Using Multilocus Genotypes. Proc. Natl. Acad. Sci. USA.

[50] Fernández-Cruz J., Fernández-Lόpez J. (2016). Genetic structure of wild sweet chestnut (*Castanea sativa* Mill.) populations in northwest Spain and their differences with other European stands. Conserv. Genet..

[51] Alessandri S., Ramos-Cabrer A.M., Martín M.A., Mattioni C., Pereira-Lorenzo S., Dondini L. (2022). Genetic Characterization of Italian and Spanish Wild and Domesticated Chestnut Trees. Sci. Hortic..

[52] Bini L., Gori M., Nin S., Natale R., Meacci E., Giordani E., Biricolti S. (2023). Assessing the Genetic Variability of Sweet Chestnut Varieties from the Tuscan Apennine Mountains (Italy). Agronomy.

[53] Alessandri S., Krznar M., Ajolfi D., Ramos Cabrer A.M., Pereira-Lorenzo S., Dondini L. (2020). Genetic Diversity of *Castanea sativa* Mill. Accessions from the Tuscan-Emilian Apennines and Emilia Romagna Region (Italy). Agronomy.

[54] Beghè D., Ganino T., Dall’Asta C., Silvanini A., Cirlini M., Fabbri A. (2013). Identification and characterization of ancient Italian chestnut cultivars using nuclear microsatellite markers. Sci. Hortic..

[55] Martín M.A., Mattioni C., Cherubini M., Taurchini D., Villani F. (2010). Genetic Characterisation of Traditional Chestnut Varieties in Italy Using Microsatellites (Simple Sequence Repeats) Markers. Ann. Appl. Biol..

[56] Marinoni D., Akkak A., Beltramo C., Guaraldo P., Boccacci P., Bounous G., Ferrara A.M., Ebone A., Viotto E., Botta R. (2013). Genetic and Morphological Characterization of Chestnut (*Castanea sativa* Mill.) Germplasm in Piedmont (North-Western Italy). Tree Genet. Genomes.

[57] Cavallini M., Lombardo G., Binelli G., Cantini C. (2022). Assessing the Genetic Identity of Tuscan Sweet Chestnut (*Castanea sativa* Mill.). Forests.

[58] de Meeûs T. (2018). Revisiting FIS, FST, Wahlund Effects, and Null Alleles. J. Hered..

[59] Valbuena-Carabaña M., Gil L. (2017). Centenary Coppicing Maintains High Levels of Genetic Diversity in a Root Resprouting Oak (*Quercus pyrenaica* Willd.). Tree Genet. Genomes.

[60] Vega C., Fernández V., Gil L., Valbuena-Carabaña M. (2022). Clonal Diversity and Fine-Scale Genetic Structure of a Keystone Species: *Ilex aquifolium*. Forests.

[61] Poulaki Konstantinidou G., Giannakopoulos N.-E., Pariotis I., Mystakidis E., Georgiadis C., Gounaris N., Tegopoulos K., Tsifintaris M., Georgitsi M., Galatsidas S. (2025). Genetic Diversity and Population Structure of Black Pine (*Pinus nigra* Arn.) in Mt. Athos, Northern Greece. Forests.

[62] Poljak I., Idžojtić M., Šatović Z., Ježić M., Ćurković-Perica M., Simovski B., Acevski J., Liber Z. (2017). Genetic Diversity of the Sweet Chestnut (*Castanea sativa* Mill.) in Central Europe and the Western Part of the Balkan Peninsula and Evidence of Marron Genotype Introgression into Wild Populations. Tree Genet. Genomes.

[63] Tumpa K., Šatović Z., Liber Z., Vidaković A., Idžojtić M., Ježić M., Ćurković-Perica M., Poljak I. (2022). Gene Flow between Wild Trees and Cultivated Varieties Shapes the Genetic Structure of Sweet Chestnut (*Castanea sativa* Mill.) Populations. Sci. Rep..

[64] Ćelepirović N., Novak Agbaba S., Bogunović S., Ivanković M., Kandemir G., Karija Vlahović M., Gradečki-Poštenjak M. (2025). Population Structure and Genetic Diversity of *Castanea sativa* Mill. Genotypes in the Republic of Croatia. Forests.

[65] Dakin E.E., Avise J.C. (2004). Microsatellite Null Alleles in Parentage Analysis. Heredity.

[66] Chapuis M.-P., Estoup A. (2007). Microsatellite Null Alleles and Estimation of Population Differentiation. Mol. Biol. Evol..

[67] Rodríguez-Ramilo S.T., Toro M.A., Wang J., Fernández J. (2014). Improving the inference of population genetic structure in the presence of related individuals. Genet. Res..

[68] Mattioni C., Cherubini M., Micheli E., Villani F., Bucci G. (2008). Role of domestication in shaping *Castanea sativa* genetic variation in Europe. Tree Genet. Genomes.

[69] Skender A., Kurtović M., Pojskic N., Kalamujic-Stroil B., Hadziabulic S., Gasi F. (2017). Genetic structure and diversity of European chestnut (*Castanea sativa* Mill.) populations in the western Balkans: On a crossroad between east and west. Genetika.

[70] Cuestas M.I., Mattioni C., Martín L.M., Vargas-Osuna E., Cherubini M., Martín M.A. (2017). Functional Genetic Diversity of Chestnut (*Castanea sativa* Mill.) Populations from Southern Spain. For. Syst..

[71] Lusini I., Velichkov I., Pollegioni P., Chiocchini F., Hinkov G., Zlatanov T., Cherubini M., Mattioni C. (2014). Estimating the Genetic Diversity and Spatial Structure of Bulgarian *Castanea sativa* Populations by SSRs: Implications for Conservation. Conserv. Genet..

[72] Petit R.J., Hampe A. (2006). Some evolutionary consequences of being a tree. Annu. Rev. Ecol. Evol. Syst..

[73] Singh M., Sodhi K.K., Paliwal A., Sharma S., Randhawa G. (2021). Efficient DNA extraction procedures for processed food derivatives-A critical step to ensure quality for GMO analysis. Food Anal. Methods.

[74] Lade B.D., Patil A.S., Paikrao H.M. (2014). Efficient genomic DNA extraction protocol from medicinally important *Passiflora foetida* containing high levels of polysaccharides and polyphenols. SpringerPlus.

[75] Viljoen C.D., Booysen C., Sreenivasen Tantuan S. (2022). The suitability of using spectrophotometry to determine the concentration and purity of DNA extracted from processed food matrices. J. Food Compos. Anal..

[76] Paithankar K.R., Prasad K.S. (1991). Precipitation of DNA by polyethylene glycol and ethanol. Nucleic Acids Res..

[77] Arbeli Z., Fuentes C.L. (2007). Improved purification and PCR amplification of DNA from environmental samples. FEMS Microbiol. Lett..

[78] Hedman J., Rådström P., Wilks M. (2013). Overcoming inhibition in real-time diagnostic PCR. PCR Detection of Microbial Pathogens.

[79] United Nations Office on Drugs and Crime (2016). Best Practice Guide for Forensic Timber Identification.

[80] Capo L.F.M., Degen B., Blanc-Jolivet C., Tysklind N., Cavers S., Mader M., Meyer-Sand B.R.V., Paredes-Villanueva K., Honorio Conorado E.N., García-Dávila C.R. (2024). Timber Tracking of *Jacaranda copaia* from the Amazon Forest Using DNA Fingerprinting. Forests.

