# Supplementary material for: Genetic Diversity in Coppice Chestnut Forests in Central Italy and Potential Use of SSR-Based Timber Traceability
Source: Plants (Basel). 2026 Jul 2;15(13):2066. doi: 10.3390/plants15132066 (PMC13363844; doi:10.3390/plants15132066)
Supplement: Supplementary file 1 [file plants-15-02066-s001.zip › Supplementary Figures.pdf]

Supplementary Figures

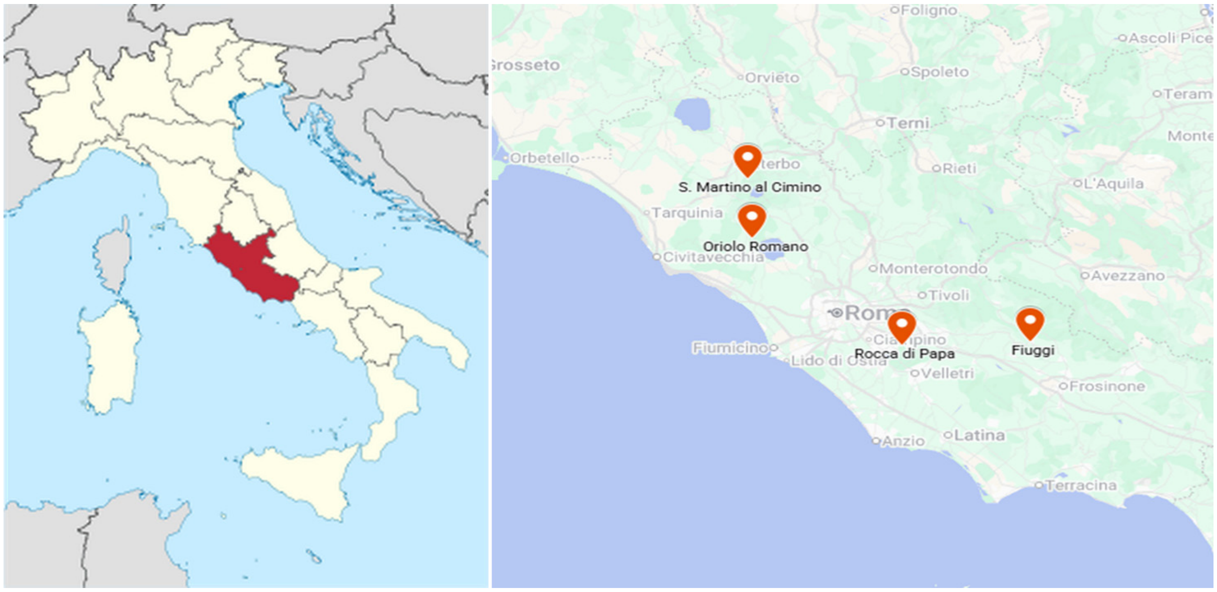

Figure S1. Map showing the four locations of coppice chestnut forests in the selected areas of the Lazio region considered in this study.

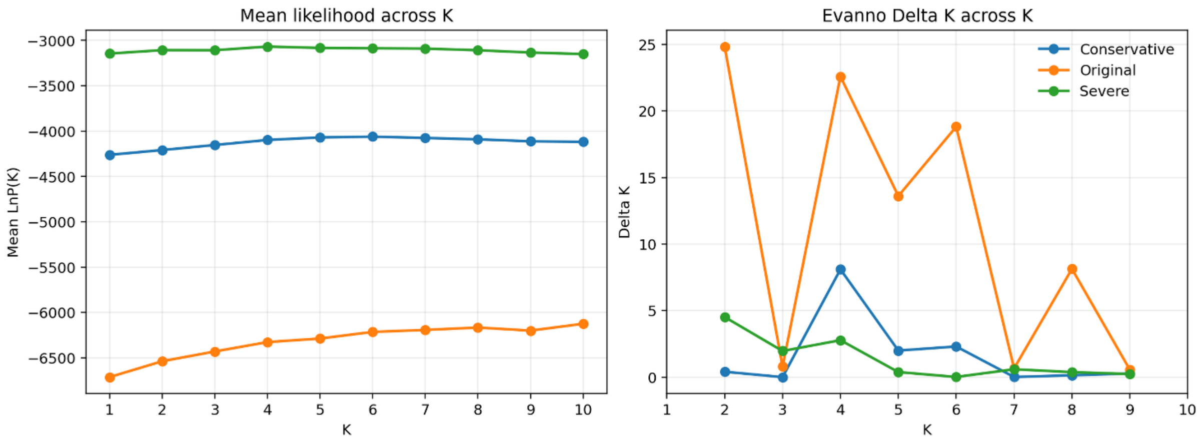

Figure S2. Mean LnP(K) and Evanno ΔK across the original, conservative, and severe post-COLONY datasets. The original dataset shows the strongest ΔK peak at K = 2, the conservative dataset peaks at K = 4, and the severe dataset shows the weakest overall signal.

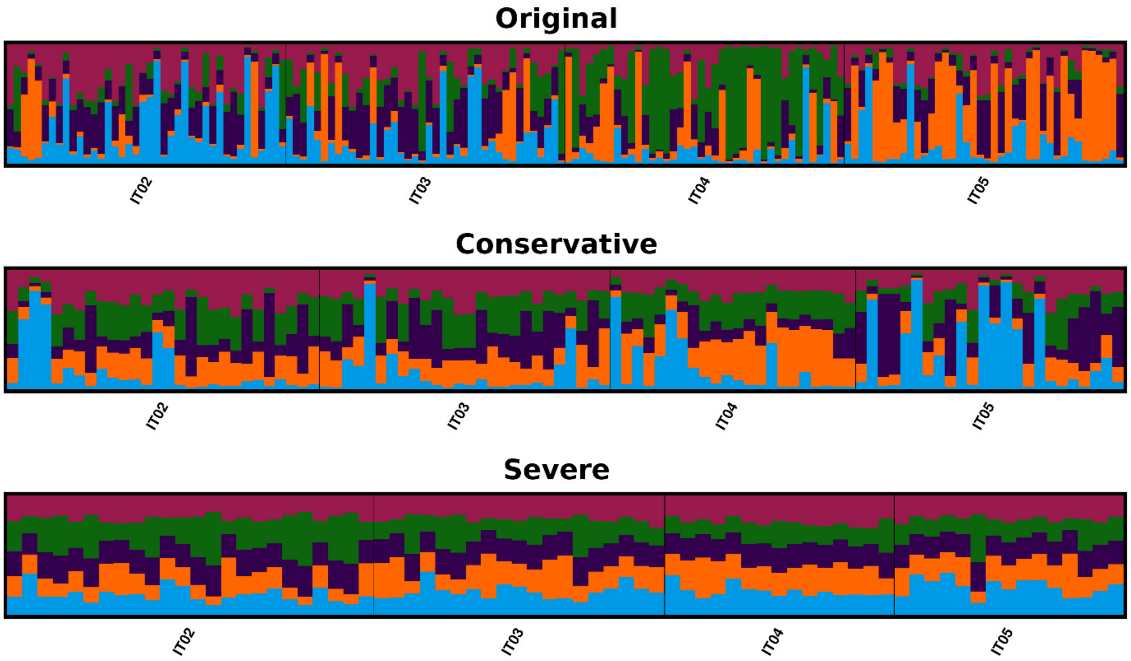

Figure S3. CLUMPAK/DISTRUCT ancestry barplots at K = 5 for the original, conservative, and severe datasets. Panels were assembled from the original CLUMPAK/DISTRUCT export files for the major mode after CLUMPP alignment. Relative to K = 4, this solution introduces finer subdivision but does not improve biological interpretability or assignment sharpness.

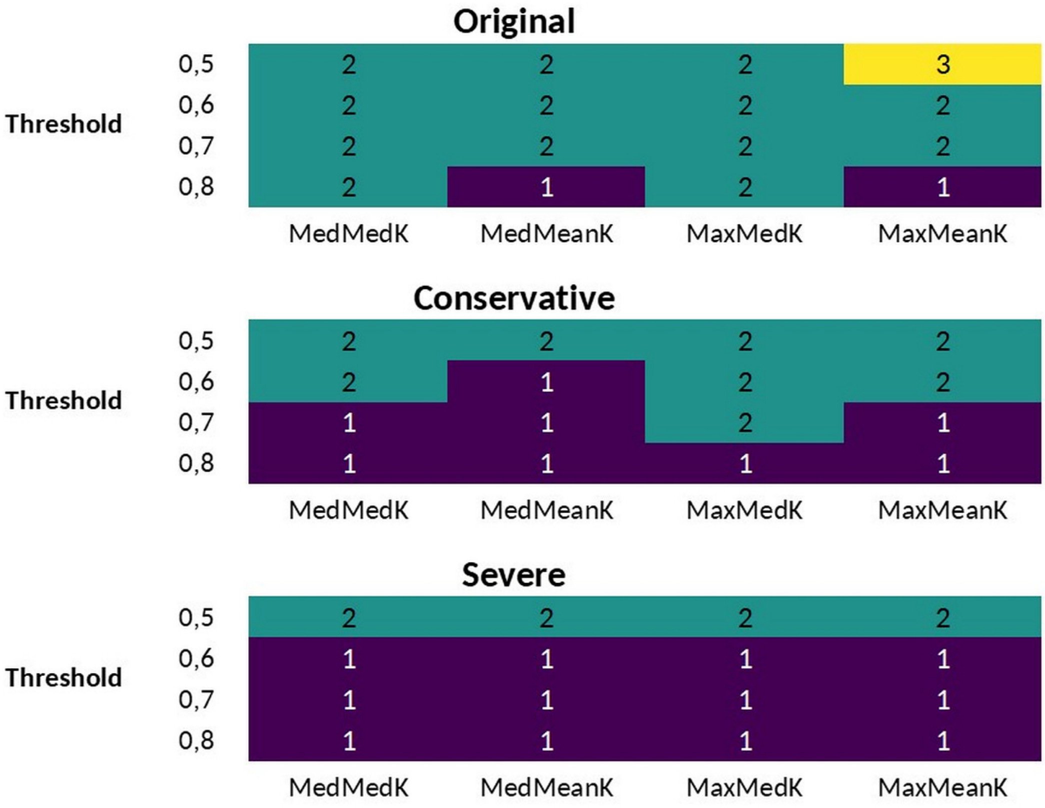

Figure S4. Custom heat map summarizing Puechmaille-selected K values across thresholds (0.5–0.8) for the original, conservative, and severe datasets, based on STRUCTURE SELECTOR output tables. Cell values indicate the K selected by each estimator (MedMedK, MedMeanK, MaxMedK, and MaxMeanK) at each threshold.

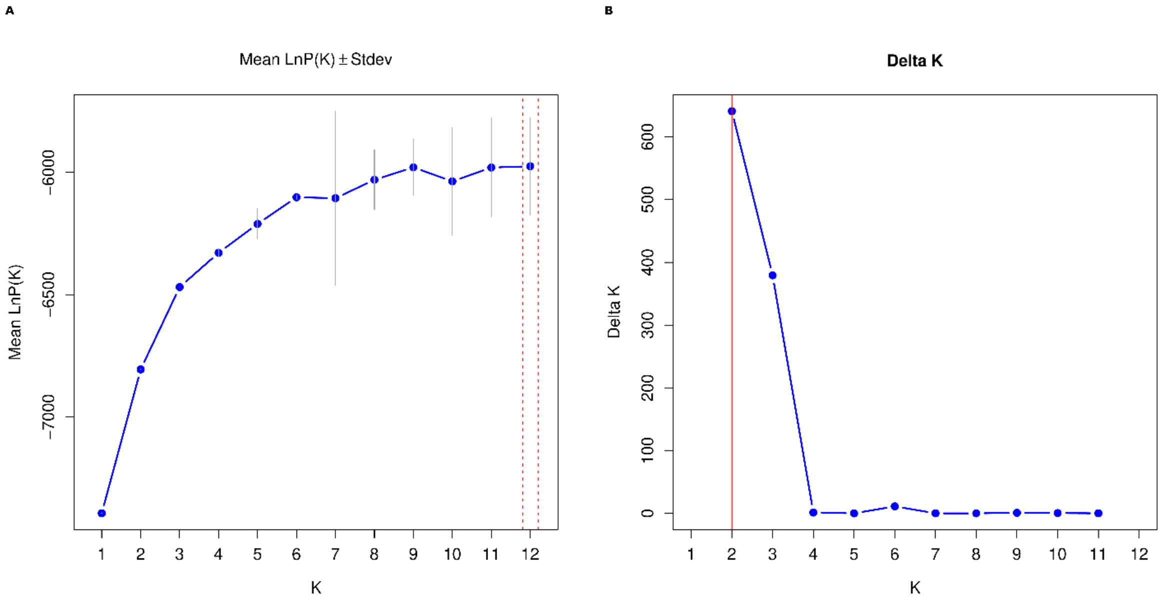

Figure S5. STRUCTURE SELECTOR summary for the 12-population dataset. Panel A shows mean LnP(K) across K = 1-12; panel B shows the Evanno Delta K statistic. The strongest support occurred at K = 2, with a secondary peak at K = 3.

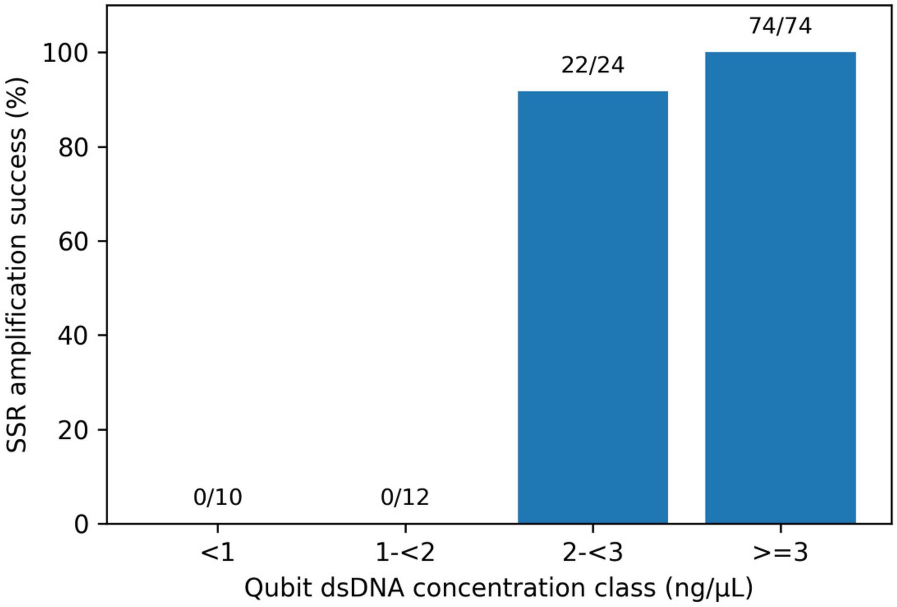

Figure S6. SSR amplification success across Qubit dsDNA concentration classes in wood extracts. Numbers above bars indicate successful amplifications/total extracts within each class.

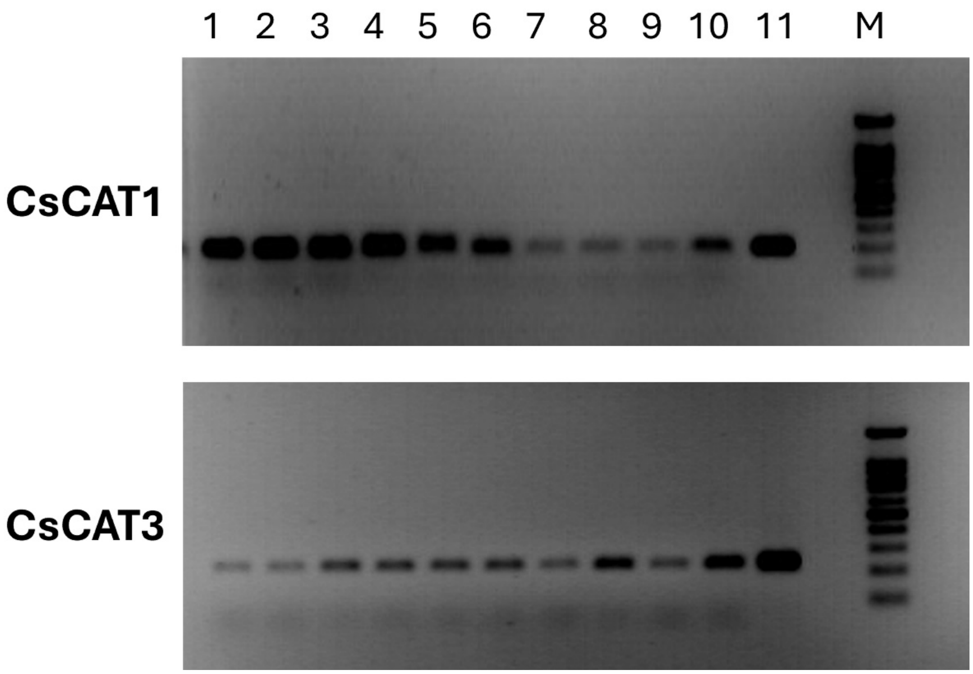

Figure S7. Agarose gel (1.2%) electrophoresis of PCR amplification products obtained from DNA extracted from woody tissue of different origin (samples 1-10) and from leaf tissue (sample 11) used as a control. Lanes 1-3: Lazio, Rocca di Papa; lanes 4-5: Calabria; lanes 6-7: France; lanes 8-10: Lazio, San Martino al Cimino; lane 11: control leaf sample; M: molecular marker (100 bp DNA ladder).

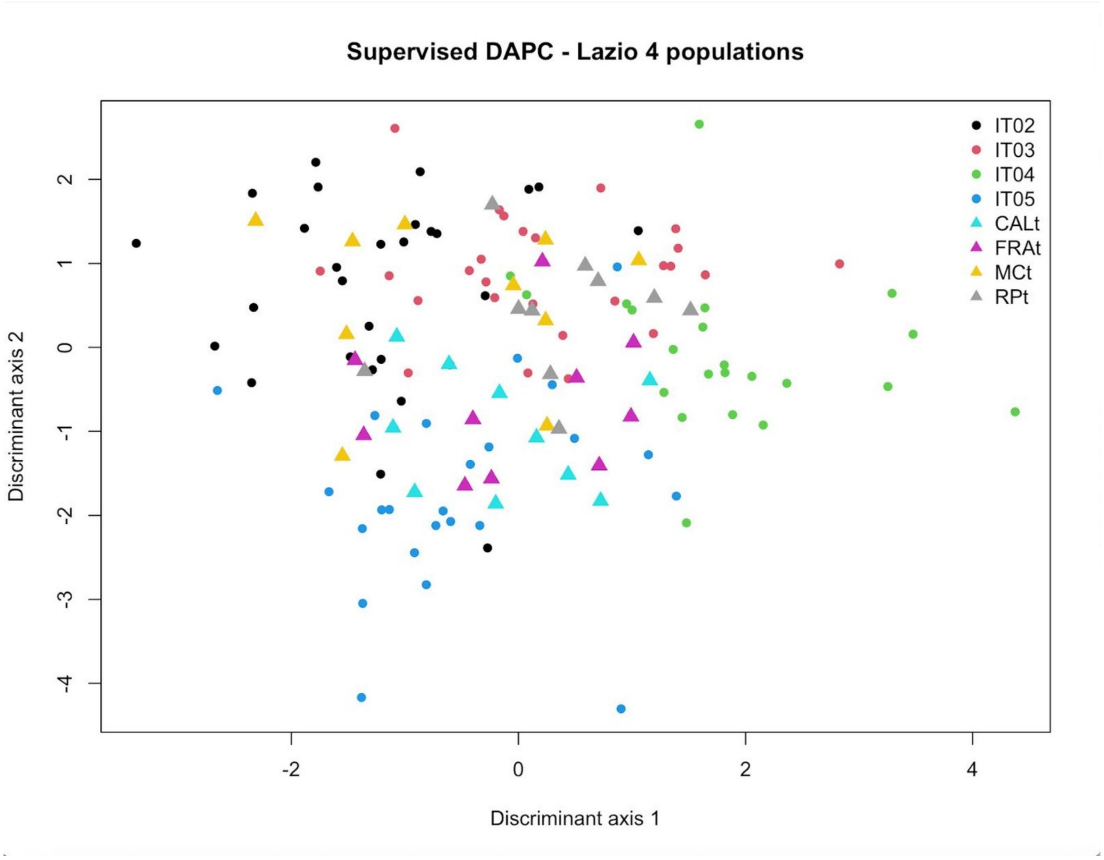

Figure S8. Supervised DAPC of the Lazio seven-locus assignment dataset. Reference individuals were used to fit the DAPC model, whereas timber samples were projected a posteriori.

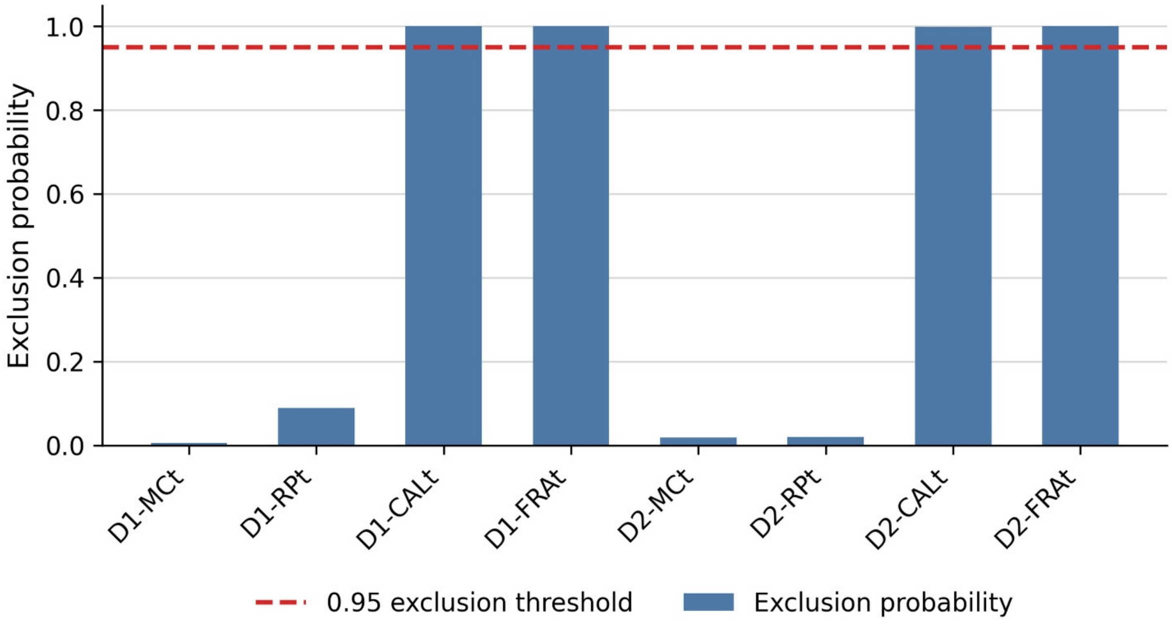

Figure S9. GDA_NT exclusion probabilities from the supplementary sawmill-level assignment analyses. The dashed horizontal line indicates the 95% exclusion threshold. D1 = Dataset 1; D2 = Dataset 2.

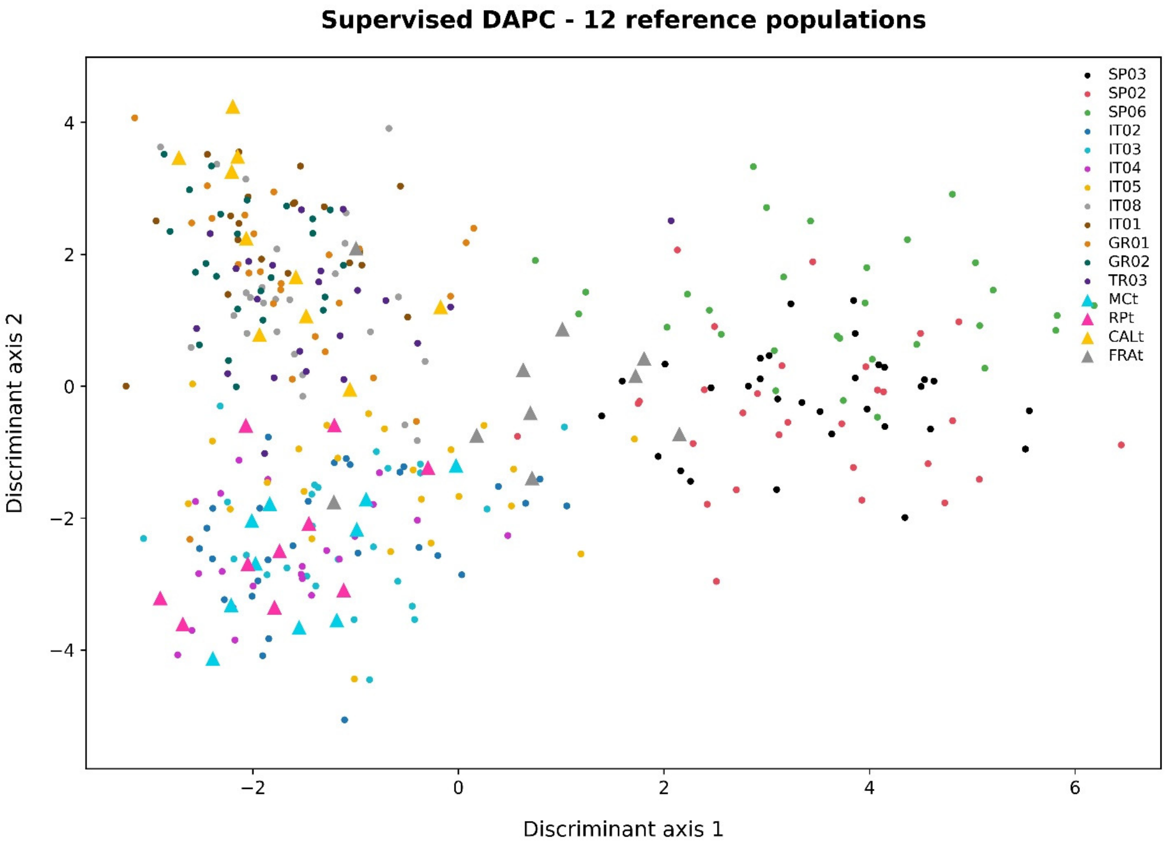

Figure S10. Supervised DAPC of the European/Mediterranean five-locus assignment dataset. Reference individuals were used to fit the DAPC model, whereas timber samples were projected a posteriori.
