# Supplementary material for: Genetic Diversity in Coppice Chestnut Forests in Central Italy and Potential Use of SSR-Based Timber Traceability
Source: Plants (Basel). 2026 Jul 2;15(13):2066. doi: 10.3390/plants15132066 (PMC13363844; doi:10.3390/plants15132066)
Supplement: Supplementary file 1 [file plants-15-02066-s001.zip › Supplementary Tables_def.pdf]

Supplementary Tables

Table S1. Characteristics of the 12 SSR loci used in this study.

Locus		Forward and Reverse primers	Product size (bp)	Repeat Motif	LG	Ta	Dye color	Reference
CsCAT1	F	GAGAATGCCCACTTTTGCA	190-224	$(TG)_5$ TA $(TG)_{24}$	8	55	6-FAM	Marinoni et al. [29]
	R	GCTCCCTTATGGTCTCG						
CsCAT2	F	GTAACTTGAAGCAGTGTGAAC	200-233	$(AG)_{16}$	10	57	HEX	Marinoni et al. [29]
	R	CGCATCATAGTGAGTGACAG						
CsCAT3	F	CACTATTTTATCATGGACGG	208-258	$(AG)_{20}$	12	53	TAMRA	Marinoni et al. [29]
	R	CGAATTGAGAGTTCATACT						
CsCAT6	F	AGTGCTCGTGGTCAGTGAG	158-198	$(AC)_{24}$ AT $(AC)_4$	1	50	TAMRA	Marinoni et al. [29]
	R	CAACTCTGCATGATAAC						
CsCAT14	F	CGAGGTTGTTGTTCATCATTAC	133-164	$(CA)_{22}$	2	55	HEX	Marinoni et al. [29]
	R	GATCTCAAGTCAAAAGGTGTC						
CsCAT16	F	CTCCTTGACTTTGAAGTTGC	125-147	$(TC)_{20}$	6	52	6-FAM	Marinoni et al. [29]
	R	CTGATCGAGAGTAATAAAG						
CsCAT17	F	TTGGCTATACTTGTTCTGCAAG	133-162	$(CA)_{19}$ A$(CA)_2$ AA$(CA)_3$	2	59	HEX	Marinoni et al. [29]
	R	GCCCCATGTTTTCTTCCATGG						
CsCAT34	F	TGAGCAAGGATGGATGATGAG	168-188	$(GT)_{23}$	-	58	6-FAM	Marinoni et al. [29]
	R	GGTGGTCATCATGACTGCATC						
CsCAT41	F	AAGTCAGCCAACACCATATGC	186-258	$(AG)_{20}$	8	55	TAMRA	Marinoni et al. [29]
	R	CCCACTGTTCATGAGTTTCT						
EMCs25	F	ATGGGAAAATGGGTAAAGCAGTAA	140-160	$(GA)_{12}$	-	58	HEX	Buck et al. [28]
	R	AACCGGAGATAGGATTGAACAGAA						
EMCs32	F	TTCCACACTTATCTCTTAACCCAAAAA	90-116	$[AG]_{18}$	12	59	6-FAM	Buck et al. [28]
	R	CTCCGGTACGGTATTGACTTCCTT						
EMCs38	F	TTTCCCTATTTCTAGTTTGTGATG	228-270	$(AG)_{31}$	4	56	TAMRA	Buck et al. [28]
	R	ATGGCGCTTTGGATGAAC						

LG = linkage group; Ta = annealing temperature (°C).

Table S2. Private SSR alleles detected in four Lazio chestnut coppice stands in homozygous (Hom) and heterozygous (Het) states. Numbers in brackets indicate the individuals carrying each allele.

Locus	Allele	Frequency	Population	Code	Status
CsCAT14	158	0.038	San Martino al Cimino (3)	IT02	Het
CsCAT3	211	0.025	San Martino al Cimino (2)	IT02	Het
CsCAT2	197	0.025	San Martino al Cimino (2)	IT02	Het
CsCAT3	267	0.013	Rocca di Papa (1)	IT03	Het
CsCAT16	134	0.013	Rocca di Papa (1)	IT03	Het
CsCAT16	153	0.013	Rocca di Papa (1)	IT03	Het
EMCs38	235	0.013	Rocca di Papa (1)	IT03	Het
CsCAT3	189	0.013	Oriolo Romano (1)	IT04	Het
CsCAT3	228	0.088	Oriolo Romano (4)	IT04	Hom/Het
CsCAT6	158	0.038	Fiuggi (2)	IT05	Hom/Het
CsCAT34	156	0.013	Fiuggi (1)	IT05	Het
CsCAT17	154	0.013	Fiuggi (1)	IT05	Het
EMCs32	81	0.025	Fiuggi (1)	IT05	Hom

Table S3. Population-level summary of HWE-related statistics based on GenAlEx and Genepop.

Population	Loci with Ho < He	Mean Ho	Mean He	Mean Fis	Genepop significant loci
IT02	11/12	0.550	0.733	0.244	9/12
IT03	11/12	0.531	0.747	0.296	9/12
IT04	10/12	0.508	0.711	0.278	8/12
IT05	11/12	0.473	0.761	0.389	10/12

Table S4. Integrated locus-level summary from GenAlEx-style statistics and Genepop exact tests.

Locus	Mean Ho	Mean He	Mean Fis	Significant populations (Genepop)	Combined exact P
CsCAT1	0.669	0.696	0.036	1	0.079
CsCAT14	0.650	0.652	0.003	0	0.815
CsCAT3	0.525	0.795	0.336	4	7.21×10^{-17}
CsCAT16	0.619	0.704	0.107	3	1.87×10^{-10}
CsCAT2	0.456	0.827	0.448	4	5.59×10^{-20}
CsCAT6	0.631	0.782	0.191	4	4.45×10^{-5}
CsCAT34	0.400	0.715	0.421	4	1.84×10^{-13}
CsCAT17	0.794	0.814	0.025	0	0.540
CsCAT41	0.444	0.795	0.441	4	2.43×10^{-21}
EMCs32	0.250	0.662	0.631	4	9.15×10^{-24}
EMCs25	0.212	0.568	0.612	4	9.15×10^{-24}
EMCs38	0.538	0.852	0.370	4	2.98×10^{-16}

Combined exact P summarizes the overall strength of Genepop evidence across populations; values are shown in scientific notation when < 0.001.

Table S5. INEst2 summary by population. Potential null-allele candidates are reported from the nf model for conservative cross-population comparison.

Population	Best DIC model	Best DIC	Mean F (f)	Mean F (nf)	Mean b (nfb)	Top p0 loci under nf
IT02	f	2732.085	0.267	0.254	0.030	CsCAT3 (0.015); CsCAT2 (0.013); CsCAT34 (0.013)
IT03	n	2784.809	0.295	0.184	0.027	CsCAT34 (0.205); EMCs25 (0.102); EMCs32 (0.089)
IT04	n	2531.185	0.303	0.072	0.024	EMCs32 (0.252); EMCs38 (0.205); EMCs25 (0.180)
IT05	f	2782.887	0.378	0.348	0.033	EMCs32 (0.067); EMCs25 (0.067); CsCAT2 (0.025)

Table S6. COLONY summary of within-population family structure and reduced datasets for downstream sensitivity analyses.

Population	Robust FS groups	Original N	Conservative N	Severe N	Removed conservative	Removed severe
IT02	6	40	28	24	12 (30.0%)	16 (40.0%)
IT03	4	40	26	19	14 (35.0%)	21 (52.5%)
IT04	6	40	22	15	18 (45.0%)	25 (62.5%)
IT05	5	40	24	15	16 (40.0%)	25 (62.5%)

Table S7. Comparative summary of STRUCTURE SELECTOR and CLUMPAK evidence used to evaluate the three post-COLONY datasets.

Dataset	N	Delta K peak	Highest mean LnP(K)	Puechmaille support	CLUMPAK K=4 major mode	Mean maxQ at K=4	Interpretation
Original	160	K=2 (24.83)	K=10	1,2,3	19/20 (sim. 0.990)	0.762	Sensitivity dataset; strongest signal but likely kin-inflated
Conservative	100	K=4 (8.10)	K=6	1,2	11/20 (sim. 0.896)	0.500	Primary dataset; best compromise after COLONY filtering
Severe	73	K=2 (4.52)	K=4	1,2	20/20 (sim. 0.871)	0.343	Overfiltered sensitivity dataset

Table S8. Comparison between K = 4 and K = 5 in terms of CLUMPAK mode stability and assignment sharpness.

Dataset	K	Major mode size	No. of modes	Mean similarity of major mode	Mean maxQ
Original	4	19/20	1	0.99	0.762
Original	5	13/20	2	0.925	0.582
Conservative	4	11/20	3	0.896	0.5
Conservative	5	20/20	1	0.876	0.446
Severe	4	20/20	1	0.871	0.343
Severe	5	20/20	1	0.885	0.292

Table S9. Pairwise differentiation among the four coppice chestnut populations from Lazio based on Nei's unbiased genetic distance (Nei uD), Fst, standardized G'st, and Nm for the conservative dataset.

Population pair	Nei uD	Fst	G'st	Nm
IT02 vs IT03	0.0734	0.0221	0.1538	11.0552
IT02 vs IT04	0.1587	0.0321	0.2178	7.5372
IT02 vs IT05	0.1694	0.0292	0.2276	8.3156
IT03 vs IT04	0.1332	0.0310	0.1964	7.8083
IT03 vs IT05	0.1372	0.0251	0.2071	9.7174
IT04 vs IT05	0.1915	0.0337	0.2455	7.1665

Table S10. Genetic diversity of the four Lazio chestnut populations in the conservative dataset. Npa = number of private alleles; Na = mean number of alleles per locus; Ne = mean effective number of alleles; I = Shannon's information index; Ho = observed heterozygosity; He = expected heterozygosity; uHe = unbiased expected heterozygosity; F = inbreeding coefficient.

Population	N	Npa	Na	Ne	I	Ho	He	uHe	F
IT02	28	4	7.250	4.305	1.590	0.565	0.728	0.742	0.211
IT03	26	2	7.417	4.320	1.649	0.545	0.757	0.772	0.284
IT04	22	1	6.250	3.684	1.430	0.492	0.696	0.712	0.267
IT05	24	5	7.333	4.783	1.677	0.490	0.765	0.782	0.368

Table S11. Summary of STRUCTURE and CLUMPAK metrics for the 12-population dataset. Panel A reports K-specific LnP(K), ΔK, and major-mode stability. Panel B reports the Puechmaille estimators across thresholds.

Panel A. K-specific model-selection and stability metrics.

K	Reps	Mean LnP(K)	Stdev LnP(K)	Delta K	Major mode runs	Mean similarity
1	20	-7394.285	0.264		20/20	1.000
2	20	-6805.785	0.392	640.987	20/20	0.999
3	20	-6468.805	0.519	379.414	20/20	0.997
4	20	-6328.580	15.899	1.385	20/20	0.901
5	20	-6210.380	62.619	0.147	15/20	0.971
6	20	-6101.355	10.034	11.236	15/20	0.992
7	20	-6105.070	356.002	0.221	16/20	0.987
8	20	-6030.030	122.644	0.195	16/20	0.913
9	20	-5978.920	114.235	0.950	14/20	0.872
10	20	-6036.360	220.633	0.517	18/20	0.880
11	20	-5979.755	202.688	0.254	17/20	0.899
12	20	-5974.595	198.465		18/20	0.873

Panel B. Puechmaille estimator summaries across thresholds

Threshold	MedMedK	MedMeanK	MaxMedK	MaxMeanK
0.5	7	7	7	7
0.6	7	5	7	6
0.7	6	4	7	4
0.8	5	3	6	4

Table S12. Genetic diversity parameters of the four Lazio coppice chestnut populations and the eight European populations used for comparison, calculated from five SSR loci. NI = number of individuals; Npa = number of private alleles; Na = number of alleles per locus; Ne = effective number of alleles; I = Shannon's information index; Ho = observed heterozygosity; He = expected heterozygosity; uHe = unbiased expected heterozygosity; F = inbreeding coefficient; Ar = allelic richness.

Population	Code	NI	Na	Ne	Npa	I	Ho	He	uHe	F	Ar
Costa Atlantica	SP03	31	9.600	5.790	5.00	1.860	0.710	0.802	0.815	0.120	8.583
Castanyet	SP02	29	10.200	5.306	0.00	1.926	0.738	0.802	0.817	0.080	9.807
Hervas	SP06	30	11.000	6.001	11.00	2.010	0.640	0.824	0.838	0.227	10.063
Monti Cimini	IT02	28	8.400	4.893	0.00	1.739	0.593	0.749	0.762	0.195	8.089
Rocca di Papa	IT03	26	8.800	4.524	1.00	1.764	0.600	0.770	0.785	0.176	8.353
Oriolo Romano	IT04	22	7.800	3.866	0.00	1.497	0.518	0.704	0.720	0.198	6.667
Fiuggi	IT05	24	8.400	5.536	0.00	1.840	0.617	0.797	0.814	0.265	8.228
Villar Pellice	IT08	26	12.200	6.364	8.00	2.029	0.746	0.809	0.825	0.076	11.013
Madonie	IT01	20	7.600	3.917	0.00	1.548	0.600	0.705	0.723	0.135	7.600
Holomontas	GR01	26	8.400	4.135	0.00	1.581	0.608	0.704	0.718	0.154	7.751
Hortiatis	GR02	24	7.600	3.440	1.00	1.420	0.633	0.639	0.653	0.022	7.201
Hopa	TR03	23	9.400	5.151	5.00	1.852	0.539	0.792	0.810	0.313	9.129
Mean			9.117	4.910	2.58	1.755	0.628	0.758	0.775	0.163	8.540

Table S14. Summary of DNA quantity and quality in wood and leaf extracts. Values are reported as median (interquartile range).

Material	n	NanoDrop DNA (ng/µL)	Qubit dsDNA (ng/µL)	NanoDrop/Qubit ratio	A260/A280	A260/A230
Wood extracts	120	20.3 (15.2-26.9)	3.6 (2.7-4.6)	6.38 (5.03-7.53)	2.22 (2.01-2.45)	1.13 (1.01-1.31)
Leaf extracts	16	80.6 (67.2-108.5)	57.0 (48.7-73.8)	1.44 (1.37-1.50)	1.69 (1.64-1.73)	1.75 (1.60-1.85)

Table S15. SSR amplification success in independent wood DNA extractions. Each timber source included 10 timber samples and three independent extractions per sample. Successful extracts indicate independent DNA extractions that generated scorable SSR-PCR profiles.

Timber source	Successful extracts	Total extracts	Success rate (%)
MCt	23/30	30	76.7
RPt	24/30	30	80.0
CALt	25/30	30	83.3
FRAt	24/30	30	80.0
**Overall**	**96/120**	**120**	**80.0**

Table S16. DAPC reference-sample confusion matrices and individual timber assignments for the Lazio and European/Mediterranean datasets. Full individual posterior probabilities are available as a separate spreadsheet. D1 = Dataset 1; D2 = Dataset 2.

Dataset	Timber source	Individual assignment counts	Posterior summary
DAPC D1	MCt	IT02=5; IT03=2; IT04=1; IT05=2	mean max posterior=0.796
DAPC D1	RPt	IT03=8; IT04=1; IT05=1	mean max posterior=0.630
DAPC D1	CALt	IT03=2; IT04=1; IT05=7	mean max posterior=0.673
DAPC D1	FRAt	IT02=1; IT03=1; IT04=3; IT05=5	mean max posterior=0.690
DAPC D2	MCt	IT02=6; IT03=1; IT04=2; IT05=1	Lazio=0.999; Spain/Iberia=0.000; Other Italy=0.001; Eastern Mediterranean=0.000
DAPC D2	RPt	IT03=4; IT04=3; IT05=3	Lazio=0.958; Spain/Iberia=0.000; Other Italy=0.037; Eastern Mediterranean=0.005
DAPC D2	CALt	GR01=2; IT01=5; IT04=1; IT08=2	Lazio=0.079; Spain/Iberia=0.001; Other Italy=0.625; Eastern Mediterranean=0.296
DAPC D2	FRAt	GR01=2; IT05=1; SP02=5; SP03=1; TR03=1	Lazio=0.170; Spain/Iberia=0.572; Other Italy=0.011; Eastern Mediterranean=0.248

Table S17. STRUCTURE individual Q-values and sawmill-level summaries for the two reference-based assignment datasets. Q-values were averaged across 20 independent runs before summarization. D1 = Dataset 1; D2 = Dataset 2.

Dataset	Timber source	Highest-Q assignment counts	Mean Q summary
STRUCTURE D1	MCt	IT02=8; IT03=1; IT05=1	IT02=0.281; IT03=0.244; IT04=0.245; IT05=0.230
STRUCTURE D1	RPt	IT03=6; IT04=4	IT02=0.239; IT03=0.269; IT04=0.251; IT05=0.241
STRUCTURE D1	CALt	IT02=2; IT03=6; IT04=1; IT05=1	IT02=0.248; IT03=0.277; IT04=0.224; IT05=0.251
STRUCTURE D1	FRAt	IT02=1; IT03=5; IT04=1; IT05=3	IT02=0.249; IT03=0.271; IT04=0.229; IT05=0.250
STRUCTURE D2	MCt	IT02=6; IT03=1; IT04=2; IT05=1	Lazio=0.721; Spain/Iberia=0.084; Other Italy=0.079; Eastern Mediterranean=0.116
STRUCTURE D2	RPt	IT03=6; IT04=3; IT08=1	Lazio=0.672; Spain/Iberia=0.091; Other Italy=0.106; Eastern Mediterranean=0.132
STRUCTURE D2	CALt	GR01=2; IT01=4; IT04=1; IT08=3	Lazio=0.227; Spain/Iberia=0.102; Other Italy=0.451; Eastern Mediterranean=0.221
STRUCTURE D2	FRAt	GR01=1; GR02=1; IT02=2; IT04=1; SP02=5	Lazio=0.301; Spain/Iberia=0.299; Other Italy=0.140; Eastern Mediterranean=0.261

Table S18. GDA_NT individual assignment results for each timber sample, including -log10 genotype likelihood, assignment score, and exclusion probability. Compatible indicates that the assigned reference population was not excluded at the 95% threshold. D1 = Dataset 1; D2 = Dataset 2.

Dataset	Timber source	Individual assignment/exclusion summary
GDA_NT D1 individual	MCt	Cimini_POP_1=7; OrioloR_POP_3=2; RPapa_POP_2=1; compatible=10/10
GDA_NT D1 individual	RPt	RPapa_POP_2=10; compatible=10/10
GDA_NT D1 individual	CALt	RPapa_POP_2=5; Fiuggi_POP_4=4; OrioloR_POP_3=1; compatible=0/10
GDA_NT D1 individual	FRAt	Fiuggi_POP_4=4; Cimini/RPapa/Oriolo=2 each; compatible=0/10
GDA_NT D2 individual	MCt	IT02=6; IT03=1; IT04=2; IT05=1; compatible=10/10
GDA_NT D2 individual	RPt	IT03=7; IT04=3; compatible=10/10
GDA_NT D2 individual	CALt	IT01=4; IT08=3; GR01=2; IT04=1; compatible=8/10; Lazio assignment excluded
GDA_NT D2 individual	FRAt	SP02=3; GR01=2; IT02=2; IT03/IT04/IT05=1 each; compatible=1/10

Table S19. Supplementary GDA_NT sawmill-level group assignment for the two assignment datasets. This group-level analysis was used only as a consistency check and was not considered the primary measure of traceability performance. D1 = Dataset 1; D2 = Dataset 2.

Dataset	Timber source	Assigned population	Exclusion probability	Interpretation
Dataset 1	MCt	Cimini_POP_1 / IT02	0.006	supported
Dataset 1	RPt	RPapa_POP_2 / IT03	0.089	supported
Dataset 1	CALt	Fiuggi_POP_4 / IT05	1.000	excluded; relative affinity only
Dataset 1	FRAt	Fiuggi_POP_4 / IT05	1.000	excluded; relative affinity only
Dataset 2	MCt	Italy_POP_4 / IT02	0.018	supported
Dataset 2	RPt	Italy_POP_5 / IT03	0.020	supported
Dataset 2	CALt	Italy_POP_9 / IT01	0.999	excluded; relative affinity only
Dataset 2	FRAt	Spain_POP_2 / SP02	1.000	excluded; relative affinity only
